# Incorporation of tetanus-epitope into virus-like particles achieves vaccine responses even in older recipients in models of psoriasis, Alzheimer’s and cat allergy

**DOI:** 10.1038/s41541-017-0030-8

**Published:** 2017-10-23

**Authors:** Andris Zeltins, Jonathan West, Franziska Zabel, Aadil El Turabi, Ina Balke, Stefanie Haas, Melanie Maudrich, Federico Storni, Paul Engeroff, Gary T. Jennings, Abhay Kotecha, David I Stuart, John Foerster, Martin F. Bachmann

**Affiliations:** 1 Latvian Biomedical Research & Study Centre, Ratsupites iela 1, Riga, LV 1067 Latvia; 2 Medical School University of Dundee, Dundee, UK; 3 HealVax GmbH, Bahnhofstrasse, 138808 Pfäffikon Switzerland; 4 HypoPet AG, c/o Universität Zürich, Moussonstrasse 2, 8044 Zürich, Switzerland; 5 Saiba GmbH, Alte Tösstalstr. 20, 8487 Rämismühle, Switzerland; 6 The Jenner Institute, University of Oxford, Oxford, UK; 7 Division of Structural Biology, University of Oxford, Oxford, UK; 8 Immunology, RIA, Inselspital, University of Bern, Bern, Switzerland

## Abstract

Monoclonal antibodies are widely used to treat non-infectious conditions but are costly. Vaccines could offer a cost-effective alternative but have been limited by sub-optimal T-cell stimulation and/or weak vaccine responses in recipients, for example, in elderly patients. We have previously shown that the repetitive structure of virus-like-particles (VLPs) can effectively bypass self-tolerance in therapeutic vaccines. Their efficacy could be increased even further by the incorporation of an epitope stimulating T cell help. However, the self-assembly and stability of VLPs from envelope monomer proteins is sensitive to geometry, rendering the incorporation of foreign epitopes difficult. We here show that it is possible to engineer VLPs derived from a non human-pathogenic plant virus to incorporate a powerful T-cell-stimulatory epitope derived from Tetanus toxoid. These VLPs (termed CMV_TT_) retain self-assembly as well as long-term stability. Since Th cell memory to Tetanus is near universal in humans, CMV_TT_-based vaccines can deliver robust antibody-responses even under limiting conditions. By way of proof of concept, we tested a range of such vaccines against chronic inflammatory conditions (model: psoriasis, antigen: interleukin-17), neurodegenerative (Alzheimer’s, β-amyloid), and allergic disease (cat allergy, Fel-d1), respectively. Vaccine responses were uniformly strong, selective, efficient *in vivo*, observed even in old mice, and employing low vaccine doses. In addition, randomly ascertained human blood cells were reactive to CMV_TT_-VLPs, confirming recognition of the incorporated Tetanus epitope. The CMV_TT_-VLP platform is adaptable to almost any antigen and its features and performance are ideally suited for the design of vaccines delivering enhanced responsiveness in aging populations.

## Noninfectious disease: Virus-like particles with wide-ranging applications

A fusion of tetanus vaccine and cucumber mosaic virus produces a therapeutic that can be adapted to treat multiple inflammatory diseases. Led by John Foerster, of University of Dundee, and Martin Bachmnann, of the University of Oxford and the University of Bern, scientists were able to take the protein coat of cucumber mosaic virus and incorporate a tetanus vaccine-derived protein structure known to stimulate the immune system. In doing so, they produced a vaccine that could accept additional stimulatory proteins and generate targeted immune responses. The team tested their therapeutic on mice and found it raised antibody levels thought to be beneficial in Alzheimer’s disease. The vaccine also showed positive results in models of psoriasis and cat allergy. More research is needed; however, many patients stand to gain from more affordable treatments for inflammatory disease.

## Introduction

Vaccines are widely used prophylactically to prevent infectious disease, as well as therapeutically to alter the course of established chronic infections. While passive immunization in the form of monoclonal antibodies (“biologics”) has had a dramatic impact on non-infectious diseases, including cancer, chronic inflammatory, and neurodegenerative disease, the development of vaccines in these increasingly important therapeutical areas has only very recently been explored clinically. The term ‘therapeutical vaccine’ has been used to denote vaccines aiming at blocking an endogenous molecular pathway in order to alter the course of an already established non-infectious condition. This approach represents a potentially attractive public health option as it could offer an affordable alternative to several mAb therapies (chapter 54 in ref. 1). Examples include vaccines against angiotensin to treat hypertension,^2,3^ TNFα to treat arthritis,^4^ IFNα to treat lupus,^5^ or IL-1β to treat diabetes.^6^ Such vaccines have been designed using a variety of strategies, including DNA-vaccination, coupling to non-specific adjuvants such as keyhole limpet hemocyanin (KLH), or virus-like particles (VLP, reviewed in^7^). However, inconsistent neutralizing capacity of vaccine-induced target-specific antibodies has often been found limiting. This may be due to a combination of factors, including the absence of good Th cell epitopes within the vaccine conferring good Th cell help to B cells,^8–10^ difficulties to bypass self-tolerance, as well as poor vaccine responses in an aging demographic due to immunosenescence.

Regarding bypassing of self-tolerance we have previously shown that the use of VLPs as antigen-carrier confers robust B cell activation, by mimicking the repetitive three-dimensional scaffold common to viral intruders.^7,11,12^ Building on this work, we here sought to explore whether incorporation of a strong T-cell epitope might deliver enhanced T cell help to B cells even under limiting conditions, such as aged vaccine recipients. The T-cell epitope derived from the tetanus toxin (TT) is uniquely capable of delivering such intrinsic immuno-boosting, since pre-existing T cell memory to this epitope is near universal in humans and has been used as such in vaccines.^13,14^ However, internal genetic fusion of this epitope to a viral envelope protein has not been reported and is not trivial since the capacity for self-assembly of icosahedral particles from VLP monomer proteins is highly sensitive to altered geometry. The search for suitable parent virus shells is further complicated by additional constraints required to obtain a platform adaptable to clinical applications including the avoidance of human pathogenic virus species, as this may perturb classical vaccination programs or interfere with diagnostics. Furthermore, selection of a sufficiently large VLP-monomer protein would increase the overall number of additional potential Th cell epitopes derived from the VLP itself.

Here we demonstrate that VLPs derived from Cucumber Mosaic Virus can be engineered to incorporate an internally fused Tet-epitope. Vaccines based on the resultant VLPs (CMV_TT_) are stable, immunogenic, and elicit strong responses even under limiting conditions. In terms of safety of plant virus-derived VLP, a recent clinical trial employing a plant-derived VLP-vaccine to treat malaria did not identify limiting safety aspects.^15^ Cucumber mosaic virus is a widely distributed virus with no toxicity to humans reported to date. In addition, there are several studies using CMV-derived vaccines in mammals including pigs, without identifying toxicity.^16^ CMV_TT_ can easily be linked to any antigen of choice via chemical cross-linking. As such, this new technology should significantly increase the utility of vaccine approaches both for the efficient targeting of signaling pathways in vivo, as well as for other clinical applications.

## Results and discussion

### Construction of a stable VLP containing a universal T cell epitope

**Fig. 1:**
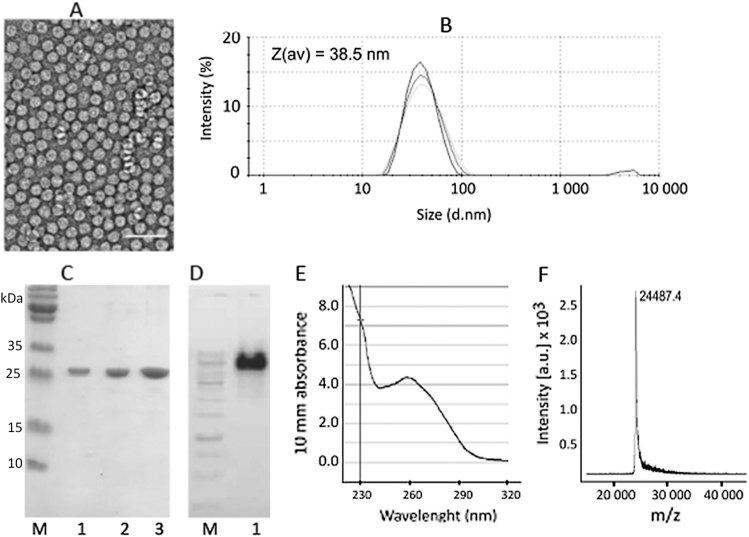
Properties of CMV_TT_ virus-like particles. **a** Electron microscopy image of purified VLPs incorporating the universal T-cell epitope (Gln Tyr Ile Lys Ala Asn Ser Lys Phe Ile Gly Ile Thr Glu) derived from Tetanus toxin. VLP sample (1.5 mg/ml) was adsorbed on carbon formvar-coated copper grids and were negatively stained with 1% uranyl acetate aqueous solution. The grids were examined using a JEM-100C electron microscope (JEOL, Tokyo, Japan) at an accelerating voltage of 80 kV. White bar corresponds to 100 nm. Particle size was found between 26 and 28 nm. **b** Dynamic light scattering (DLS) analysis of CMV_TT_. Sample VLP solution (1 mg/ml) was analyzed on a Zetasizer Nano ZS instrument (Malvern Instruments Ltd, UK). The results of three measurements were analyzed by DTS software (Malvern, version 6.32). The average hydrodynamic diameter (Z(av)) of particles was found 38.5 nm. **c** SDS-PAGE analysis of CMV_TT_ VLPs. Increasing amounts of VLPs were loaded on the gel (lane 1–0.6 µg, lane 2–1.2 µg and lane 3–2.4 µg). M—protein size marker (Page Ruler Plus, Thermo Scientific). Samples were derived from the same experiment and were processed in parallel. **d** Agarose gel analysis of CMV_TT_. Lane 1 – CMV VLPs (4 µl, 1.5 mg/ml) were mixed with DNA Loading buffer (lane 1) and analyzed in 0.8% agarose /TBE buffer. M – DNA size marker (Gene Ruler, 1 kb, Thermo Scientific). Samples were derived from the same experiment and were processed in parallel. **e** UV spectroscopy. The UV spectrum of VLP solution (1 mg/ml) was recorded using a Nanodrop ND-1000 spectrophotometer (NanoDrop Technologies, Wilmington, USA). The VLP sample absorbs strongly at 260 nm which is typical for viruses and VLPs. **f** For mass spectrometric (MS) analysis CMV_TT_ VLPs (1 mg/ml) were diluted with a 3-hydroxypicolinic acid matrix solution and were spotted onto an MTP AnchorChip 400/384TF. Matrix-assisted laser desorption/ionization (MALDI)-TOF MS analysis was carried out on an Autoflex MS (Bruker Daltonik, Germany). The protein molecular mass (MM) calibration standard II (22.3–66.5 kDa; Bruker Daltonik) was used for mass determination. Obtained spectrum suggests that the first methionine is removed during the CMV coat protein synthesis in E. coli cells and the N-terminus is replaced by the TT epitope (calculated MW 24485 Da, found m/z value 24487.3 Da)

We set out to engineer a VLP particle maintaining structural stability as well as the capacity for self-assembly even after incorporation of an artificial sequence into the envelope protein monomers. In addition, in order to be adaptable to a wide range of applications, the ideal VLP would (1) be derived from a non-human parent virus, (2) retain the capacity to incorporate RNA able to serve as immuno-stimulatory TLR agonist RNA,^17,^ ^18^ and (3) exhibit a large molecular size monomer to increase the overall number of T-cell stimulatory epitopes. Based on these criteria, we screened several candidate plant virus derived VLPs harboring large (25–30 kDa) monomer blocks, almost twice the molecular weight of e.g., RNA-phage derived VLPs. Several candidate VLPs exhibited dramatically reduced or absent VLP stability upon insertion of the Tetanus-derived epitope (TT) at various sites (Suppl. Fig. 1). In contrast, replacement of the first 12 N-terminal amino acids of CMV with the TT epitope resulted in well assembled VLPs with a size of approximately 30–40 nm in diameter, as shown by electron microscopy (EM) (Fig. 1a) and dynamic light scattering (DLS) (Fig. 1b), preserving the native T = 3 icosahedral structure. SDS-PAGE analysis revealed a homogenous product of the expected size (Fig. 1c). Ethidium bromide staining (Fig. 1d) and UV spectroscopy (Fig. 1e) confirmed preserved RNA incorporation. Mass spectroscopy confirmed one major peak of the expected size (Fig. 1f). CMV_TT_-VLPs were stable for at least 12 weeks both at −20 °C or 4 °C (Suppl. Fig. 2). This was further confirmed by DLS analysis showing stability with only a low tendency to aggregate generation as indicated by a second peak with increased mean hydrodynamic size (Z (av)).

### Structure of CMV_TT_ VLPs

**Fig. 2:**
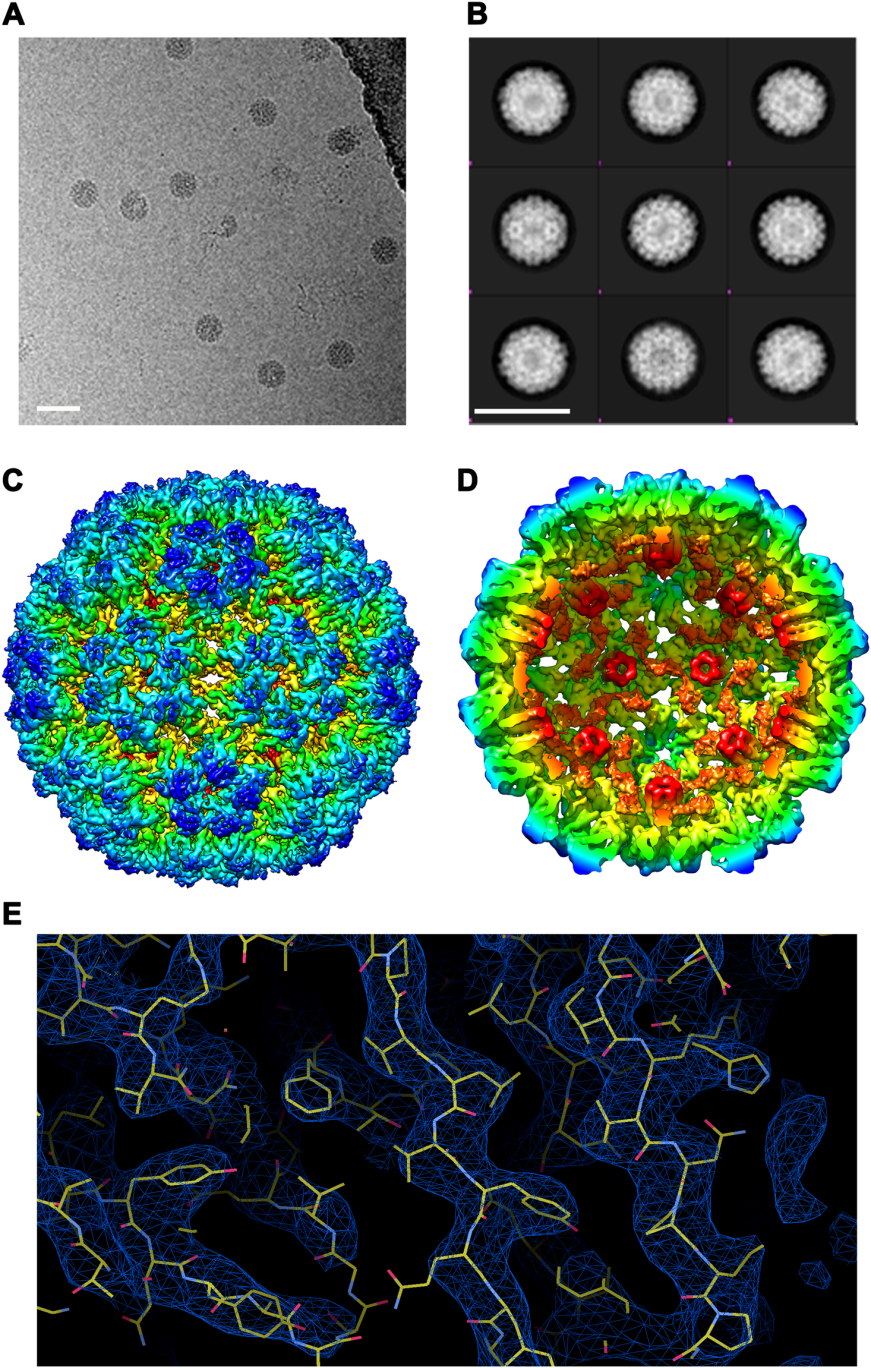
Structure resolution of engineered CMV VLPs by cryoEM. VLPs were dissolved in borate-EDTA containing buffer, vitrified and analyzed by EM using a Tecnai F30 ‘Polara’ microscope (FEI) **a** Representative 2-D view of vitrified VLP, generated from repeated imaging each field of view along the Z- axis creating a series of images (constituting a movie). From ~300 movies, 6685 particles were selected for data processing. **b** Examples of generated 2-D classification, 20 were generated based on average electron density. White bar corresponds to 50 nm. Cross sectional Selection refinement towards 3-D electron density model and an initial model fitting data to icosahedral symmetry produced 9 Å model. **c** High level refinement further improved resolution to 4.2 Å. In addition, **d** the internal view reveals ordered elements (shown in orange) are visible on the interior of the capsid shell. **e** Resolution Fitting reconstruction reveals that the obtained structure corresponds to X-ray crystal structural data

We next characterized the molecular structure of the newly generated CMV_TT_ VLPs by cryoEM. High resolution images were captured (Fig. 2a) and more than 6600 VLPs were picked for averaging, resulting in several 2D classes of VLPs (Fig. 2b); the best of these were used for 3D- classification. This allowed construction of a 9 Å model, followed by refinement to 4.2 Å (Figs. 2c, d), and fitting of the primary sequence into the structure (Fig. 2e). The structure of CMV_TT_ appeared largely identical to the parent CMV particles (RMSD 1.8 Å), particularly at the surface. Specifically, the subunit arrangement of the asymmetric unit (chains A, B and C) was identical between the parent virus and the modified VLPs. The AA dimers were arranged at the 5-fold axis and the BC dimers around the 3-fold/ pseudo 6-fold axis. Upon closer inspection there were minor differences in the spatial location of the backbone polypeptide in the capsid interior, most likely due to the lack of native viral capsid–genome interactions in the recombinant VLPs. Instead, the modified VLP exhibited additional electron density displaying 3-fold symmetry within the interior cavity (Fig. 2d, orange color), indicative of novel ordered structures, most likely reflecting tightly bound internal RNA. Interestingly, their relative location and resemblance to tightly-associated RNA species would suggest they might contribute towards stabilizing of intra-subunit associations underlying the overall stability of the particles. A further intriguing aspect of the structure is the relatively large pore size, which may allow for the exchange of naturally bound RNA by other poly-anions or other cargo.

**Fig. 3:**
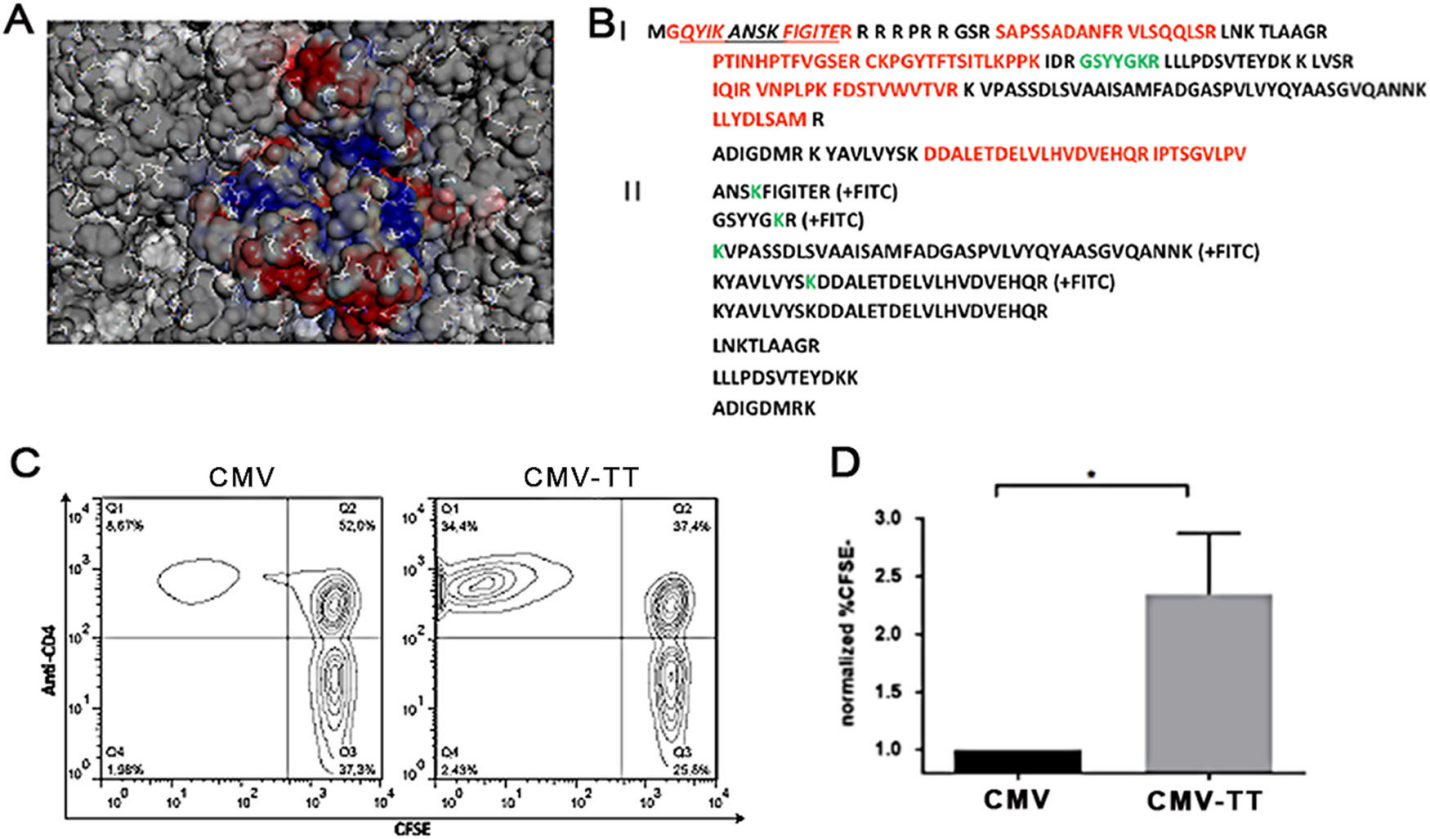
Chemical reactivity of surface exposed Lysine residues and T cell reactivity of incorporated universal T cell epitope. **a** Surface representation of cryoEM model with all lysine residues displayed in stick format, net surface charge is color coded. **b** Trypsin fingerprint analysis of CMV-Ntt830 VLPs after treatment with fluorescein isothiocyanate (FITC). **Panel I**—CMV-Ntt830 protein sequence represented in the form of trypsin peptides. Red—peptides identified after trypsin digest and mass spectrometric analysis; black—peptides not found; green—peptide identified only in the form of FITC conjugate. The Ntt830 residues are underlined; **Panel II**—CMV-Ntt830 coat protein peptides found as FITC conjugates (green Lys symbols) or partially cut by trypsin. **c,d** The universal T cell epitope in CMV_TT_ is recognized by primary human CD4^+^ T cells. **c** Human PBMCs from four individual donors were labeled with CFSE and cultured for 7 days upon stimulation with either CMV or CMV_TT_ and CFSE fluorescence was assessed by flow cytometry. Shown are representative scatterplots of CD4^+^ T cells stimulated with either CMV or CMV_TT_. **d** Normalized percentages of CFSE-CD4^+^ T cells in 4 individual donors upon CMV (black) or CMV_TT_ (gray) are displayed as mean ± SEM. *p < 0.01 in a two-tailed, unpaired student’s *t* test

A straightforward method to display antigens of choice on the surface of VLPs is through chemical coupling to surface exposed Lys. We therefore mapped all surface exposed Lys in the VLP-structure (Fig. 3a). Next, FITC conjugation to CMV_TT_, followed by trypsin-digest and MS was used to identify Lys residues functionally available for coupling. This showed that 4 of 8 tryptic peptides generated were detected with the FITC modification (Fig. 3b). One surface located Lys-containing peptide (GSYYG**K**R, green letters in Fig. 3b, Panel I) was completely modified by FITC. In all other cases, both unmodified and FITC-modified peptide forms were found. These results correspond well to the cryoEM structural model, which shows several surface located Lys to be orientated inwards thereby providing insufficient space for multiple crosslinker-VLP interactions. Ionic charge also was an efficient predictor of coupling, since two prominent surface exposed Lys (equivalent to K79 and K116 in the wild type virus) located in acidic patches did not couple to FITC, while K88 residing nearby but with different charge was efficiently modified. Taken together, these data provide an in-depth structural characterization of the novel VLP-species and confirm that ample surface-exposed lysine residues are present to allow chemical cross-linking to antigenic epitopes of choice.

### Recognition of the universal T cell epitope in CMV_TT_ by human CD4^+^ T cells

In order to test recognition of the tetanus-derived universal T cell epitope, human PBMCs were labeled with CFSE and stimulated with either wild type or CMV_TT_ followed by measurement of proliferation by assessment of the frequency of CFSE^low^ CD4^+^ T cells 7 days later. With each cell division, cellular CFSE-labeling is reduced by 50% and CFSE^low^ CD4^+^ T cells therefore represent T cells having undergone cell division. The frequency of these cells was much higher in PBMC cultures stimulated with CMV_TT_ compared to those stimulated with CMV wild type, in fact comparable to proliferation of cells stimulated with pure tetanus toxoid as positive control, thereby confirming robust recognition of the universal T cell epitope in CMV_TT_ (Fig. 3c, left upper quadrant and Fig. 3d). Therefore, incorporation of the universal T-epitope in CMV_TT_ boosts T-cell responses in randomly selected primary human T cells.

### Generation of a vaccine against psoriasis

**Fig. 4:**
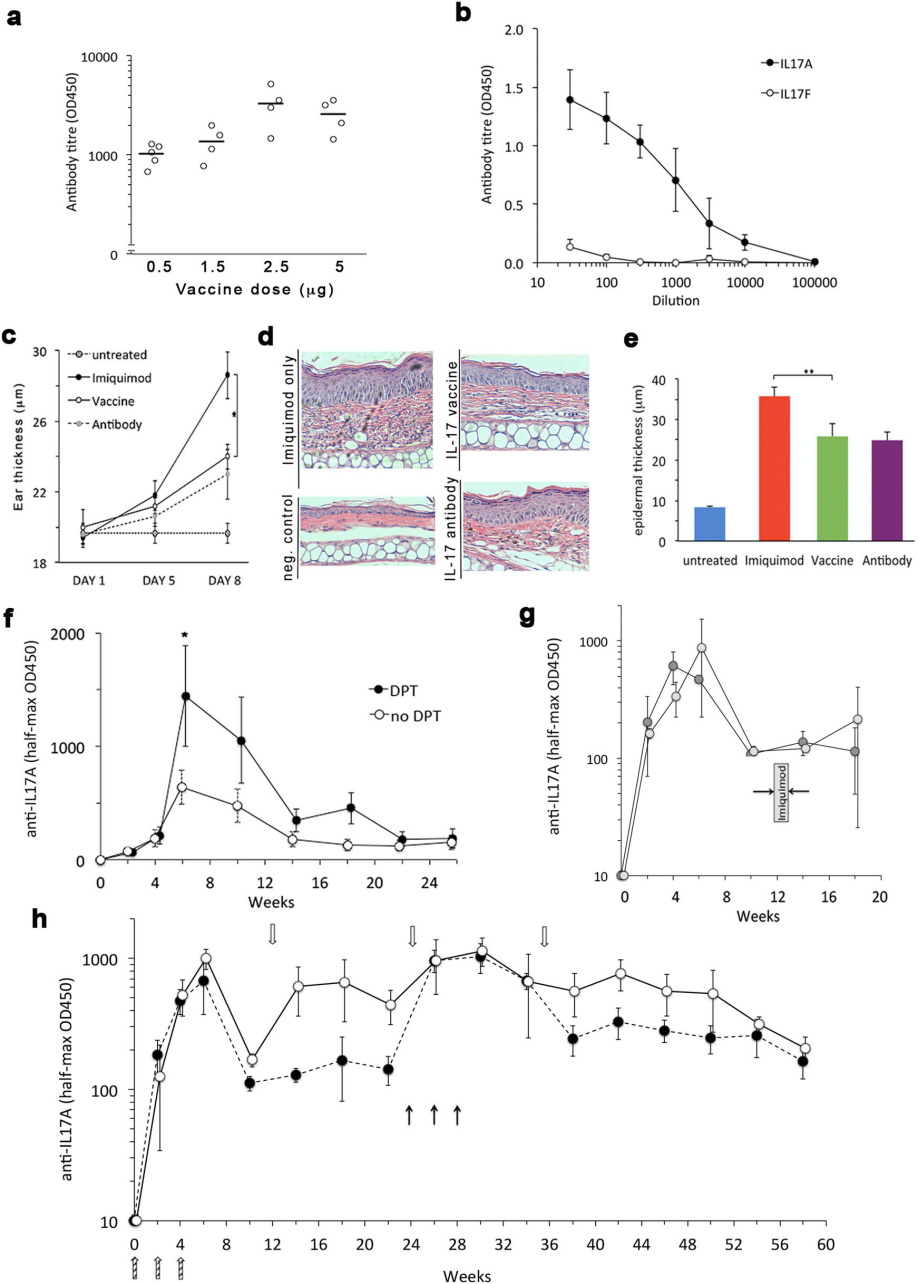
Immunogenicity and specificity of the immune response generated by vaccination with a CMV-based anti IL17A vaccine in mice. **a** Female C57B/6j mice aged 8 weeks (*n* = 4 per group) were immunized as detailed in the text and anti-IL17A IgG titers determined based on half-maximal OD450 values, as previously described.^18^ **b** Specific IgG antibody titers ELISA assays of *n* = 4 mice were performed with plating either recombinant murine IL17A (black circles) or IL17F (open circles). **c** The therapeutical effect of IL17A-vaccination on psoriasis-like disease in mice. Female C57B/6j mice (*n* = 5 per group) were vaccinated three times (day 0, 14, 28) with 50 µl (2.5 µg) of vaccine. Two weeks after the last vaccine booster, imiquimod cream was applied daily to the ears and dorsal skin (see Methods). Comparator groups were mice not receiving any treatment, non-immunized mice receiving direct anti-IL17 antibody injection, and untreated mice, as indicated in the figure. Ear thickness was determined on the days indicated. Data shown represent average ± s.d. *p < 0.05 (two-tailed T-test). **d**, representative H&E-based histology of mice shown in **c** sacrificed on day 8. **e**. Quantification of epidermal thickness in all samples. **p < 0.01. **f** The effect of tetanus-pre-vaccination on IL17A-vaccine immunogenicity. Female C57/B6j mice at young (a, 10 weeks) or advanced (b, 10 months) age received either no (open symbols) or DPT-prevaccination (closed symbols). Four weeks later, 0.5 µg of IL17A vaccine was administered on days 0,14,28 and IL17A titers determined by ELISA. *p < 0.05 (two-tailed t-test). **g** Endogenous boosting. Female C57B/6j mice (*n* = 5 per group) received triple IL17A vaccination (0,2,4 weeks) followed by no treatment (dark shaded) or one week of imiquimod treatment (light shaded) identical to that employed in Fig. 3. IL17A-IgG titers were measured by ELISA. **h** Long-term anti-IL17A titer kinetics. Female mice were vaccinated at baseline (0,2,4 hatched arrows on bottom), followed by booster vaccination either as single injection every three months (white symbols and arrows), or one single triple injection booster after six months (black symbols). Data shown are average and SEM

We next proceeded to design clinically relevant vaccine prototypes based on CMV_TT_ to explore functional performance. First, we focussed on Interleukin 17 A (IL17A), a pro-inflammatory cytokine. Antibodies targeting IL17A are highly effective in psoriasis, a skin condition affecting between 1 and 4 % in most global populations.^19^ Despite an excellent safety and efficacy profile, the high cost of IL17-targeting biologics severely restricts access to this treatment. Replacing these antibodies by vaccination against IL-17A therefore has the potential to vastly increase access to treatment for a wide range of patients (reviewed in ref. 20) as well as be useful for other conditions, as we have previously shown using an earlier VLP-based IL17 vaccine.^21^ To test the performance of a CMV_TT_ based anti-IL17A vaccine for psoriasis, we first generated a vaccine prototype by conjugation of full-length, dimeric murine IL-17A to CMV_TT_ (Figure S3). Next, mice were immunized with different doses of the IL-17 CMV_TT_ vaccine preparation, resulting in high anti-IL-17A IgG levels, after as low a dose as 0.5 µg (Fig. 4a), but not to the most highly conserved IL17 isoform, IL-17F (Fig. 4b), confirming high selectivity of the induced response.

### Preclinical efficacy of the IL17CMV_TT_

We next assessed the ability of the vaccine to reduce psoriatic disease in vivo, employing the widely used IL17-dependent imiquimod model^22^ in a direct head-to-head comparison to a high-affinity monoclonal IL17 antibody. As shown in Fig. 4c, serial measurement of ear thickness revealed an approximately 50% reduction of ear swelling in animals receiving either protective vaccination or treatment with IL17A antibody. The same was observed in ex-vivo quantification of epidermal thickness by histology (Fig. 4d/e). Efficacy of the vaccine was also equivalent to the anti-IL17 antibody when tested in dorsal skin (Figure S4). These data suggest that a CMV_TT_ based IL17A-vaccine is equipotent to a monoclonal anti-IL17A antibody in mice.

### Immunogenicity under limiting conditions

In clinical practice, eliciting an effective immune response in the vast majority of vaccine recipients can be a major challenge. In psoriasis, an ageing demographic may contribute to a sub-optimal immune responses. In order to obtain evidence whether incorporation of the TT-epitope would boost immunogenicity in poor responders, we emulated sub-optimal vaccine conditions, both by reducing the vaccine dose to the threshold required for a response (0.5 µg) and studying aged mice (10 months). Indeed, as shown in Fig. 4f, we observed an increased formation of IL17A IgG in response to vaccination in mice that had received a DPT-vaccination prior to IL17A vaccination. The same trend was independently observed in young mice (Figure S5). These data suggest that inclusion of a tetanus-derived epitope augments immunogenicity under limiting conditions.

### Absence of endogenous IL-17 boosting, reversibility, and ability to re-vaccinate

Finally, we characterized additional parameters important for clinical applications. First, high local IL17A levels in the skin during a psoriasis-flare could theoretically cause an endogenous boosting of anti-IL17A titerss. We therefore simulated a psoriasis flare by application of imiquimod cream to mice having received prior anti-IL17A vaccination. However, we did not observe any effect of this treatment on antibody titerss (Fig. 4g). Next, we determined long-term titers kinetics. In confirmation of previous experience with VLP-type therapeutical vaccines in humans, anti-IL17A IgG titerss were reversible after approximately 10 weeks (Fig. 4h). Importantly, a single 3-monthly vaccine booster shot was able to maintain IL17A IgG titerss (white symbols). Furthermore, an alternative boosting regimen applied as single triple-boost after six months was able to re-induce antibody titerss without causing high titers ‘super boosting’. We conclude that CMV_TT_-based IL17A vaccination results in potent, reversible, and re-boostable anti IL17A IgG titers in mice, devoid of endogenous boosting during psoriasis-like flares.

### Safety of IL17-vaccination

In terms of safety of an IL17-targeting vaccine approach, it is worth noting that, to date, in excess of 5000 patients have been continuously exposed to neutralizing anti-IL17 action (ixekizumab, secukinumab, brodalumab) for at least five years as part of long-term extension clinical trials as well as post-marketing. This extensive clinical dataset has not identified any delayed-onset safety aspects. This duration of IL17 neutralization by far exceeds the expected duration of anti-IL17 suppression effected by a VLP-vaccine based approach, suggesting the latter is unlikely to add safety concerns. In terms of non-target related autoimmune-issues, previous experience with VLP-based vaccines (angiotensin, IL1-targeting vaccines) has not uncovered off-target non-specific T-cell activation facilitating undesired autoimmune effects. Although clinical trials will be required to confirm safety, presently available data therefore do not indicate principle safety limitations of this approach.

### Generation of a vaccine against cat allergy

**Fig. 5:**
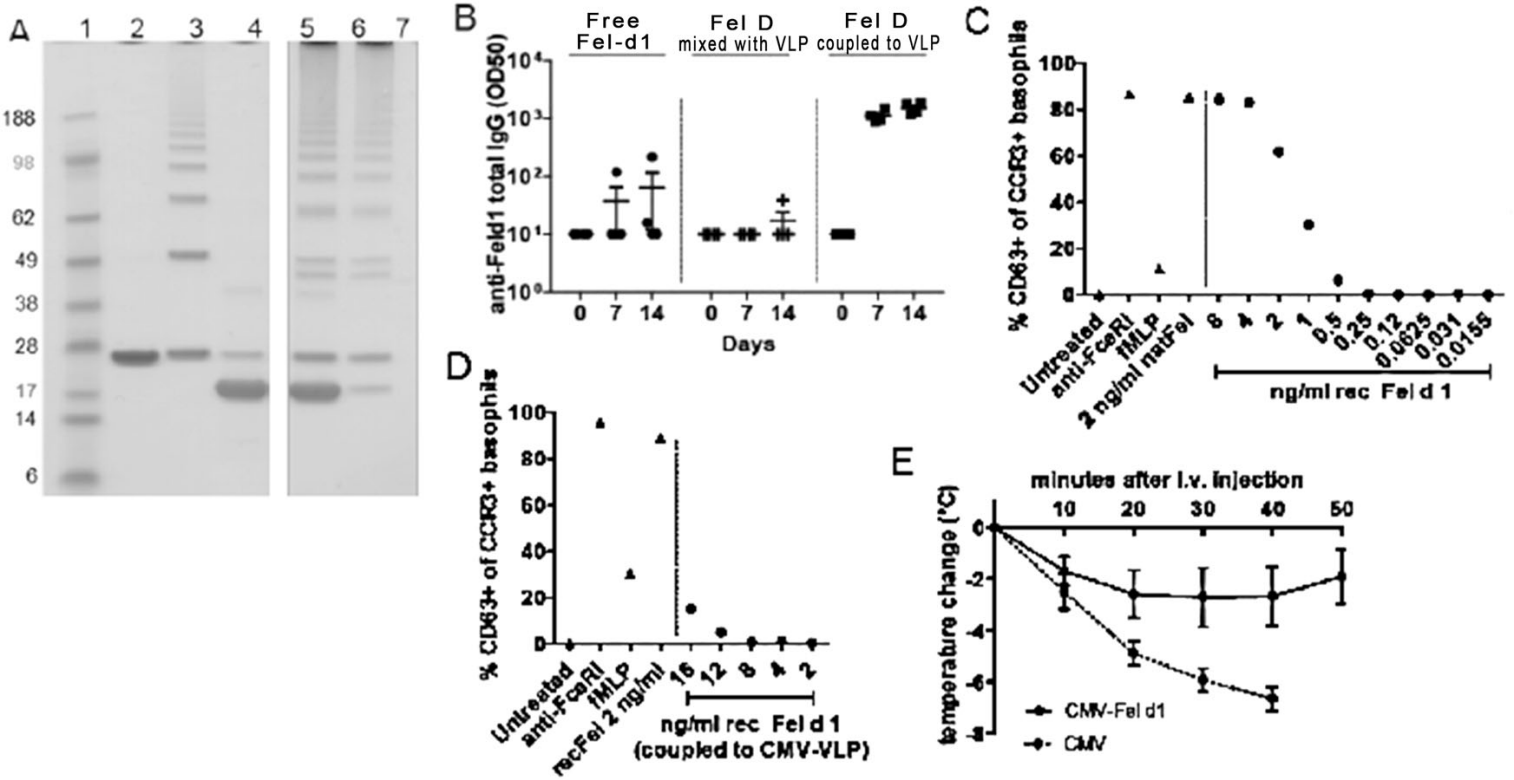
Generation of a vaccine against cat allergy. **a** Analysis of the recombinant Fel-CMV_TT_ vaccine. SDS Page (NuPage 4–12% Bis-Tris) was run under denaturing and reducing conditions. Lanes from left to right: 1-MW markers (Invitrogen, SeeBlue Prestained), 2-purified CMV VLP (~ 25 kDa coat protein monomer), 3-derivatized CMV_TT_-VLP after reaction with SMPH (bands in order of ascending size corresponding to VLP monomers, dimers, trimers etc. resulting from cross-linking), 4-purified Fel d 1 (~20 kDa), 6/7-vaccine Fel-CMV_TT_ following conjugation with Fel d 1 protein, loaded amount of VLP 5 µg and 10 µg, 9/10-Fel-CMV_TT_ after removal of uncoupled Fel d 1 by ultrafiltration, loaded total protein amount of VLP 5 µg and 10 µg. Samples were derived from the same experiment and were processed in parallel. **b** Immunogenicity assessment in Balb/C mice that received 1 µg of Fel d 1 protein in three versions: free, mixed with or coupled to CMV_TT_ intravenously on day 0. Blood was collected on days 0, 7 and 14 and analyzed for Fel d 1 specific total IgG titers (OD50). **c, d** Basophil activation test was performed with whole blood from a cat-allergic patient. Blood samples were incubated with different amounts of free recombinant Fel d 1 **c** or recombinant Fel d 1 coupled to CMV_TT_ **d**. Untreated, anti-FceRI, fMLP and natural Fel d 1 served as controls. **e** Fel d 1 allergen challenge *in-vivo*. Mice were sensitized with natural Fel d 1 and vaccinated with Fel-CMV_TT_ vaccine or CMV_TT_ VLP alone. To show protection of sensitized mice upon immunization, mice were challenged with 3 μg natural Fel d 1 in 150 μl PBS i.v. Mean temperature changes (°C) in Fel-CMV_TT_ vaccine or CMV_TT_-VLPs-vaccinated mice +/- SEM are shown (*n* = 5). After 40 min CMV_TT_-vaccinated mice were sacrificed because of severe anaphylactic symptoms

Allergy to cats is highly prevalent and affects up to 10% of the common population. It is not restricted to cat owners,^23^ and often has major secondary effects on other atopic conditions such as eczema or asthma. The currently available treatment, specific immunotherapy, lacks consistent efficacy and is fraught with safety issues. Thus, widely available effective treatment for this condition would have significant health benefits. The major allergen responsible for most allergies against cats world-wide is feline domesticus 1 (Fel d 1),^24^ a 35–39 kDa heterodimer.^25^ We have previously shown that recombinant Fel d 1 displayed on VLPs derived from the bacteriophage Qβ efficiently confers protection in a murine model of cat allergy.^26^ To extend these findings to CMV_TT_-VLPs, recombinant Fel d 1, produced as described previously,^26^ was coupled to CMV_TT_ via a small Cys containing linker^26^ (Fig. 5a). To test the immunogenicity of the vaccine, mice were immunized with Fel d 1 either soluble, mixed with CMV_TT_ particles or coupled to CMV_TT_ particles. Fel d 1 coupled to CMV_TT_ was highly immunogenic and induced a 10–100 fold stronger antibody response than free Fel d 1 or Fel d 1 mixed with VLPs (Fig. 5b), demonstrating potent immunogenicity depended on presentation by the CMV_TT_-VLP.

The main safety limitation of specific immunotherapy against cat allergy is the risk of severe anaphylactic reactions. We therefore tested whether Fel d 1 coupled to CMV_TT_ would carry a lower risk of anaphylaxis than free Fel d 1. To this end, we stimulated basophils in whole blood from an allergic human donor with either free Fel d 1 or Fel d 1 conjugated to CMV_TT_ and assessed basophil activation. As expected, free Fel d 1 induced strong basophil degranulation over a wide range of concentrations (Fig. 5c). By contrast, degranulation after stimulation with similar amounts of conjugate Fel-CMV_TT_ was greatly reduced (Fig. 5d). Thus, VLP-coupling effectively reduces the anaphylactic reactivity of this potent antigen.

The therapeutic potential of the vaccine Fel-CMV_TT_ was tested next. BALB/c mice were rendered allergic by injection of low doses of natural Fel d 1 in Alum to induce Fel d 1 specific IgE. Mice were then injected either with Fel d 1-CMV_TT_ or unconjugated CMV_TT_ as a control. Two weeks after injection, mice were re-challenged with the allergen and anaphylactic responsiveness was quantified intra-vital by serial measurement of change in body core temperature (Fig. 5e). Control mice that had received the VLP alone showed a severe drop in temperature, indicative of a strong systemic allergic reaction. In contrast, mice immunized with the vaccine Fel-CMV_TT_ were largely protected (Fig. 5e). Since desensitization to IgE mediated cat allergy ranks as a challenging test for anti-anaphylactic treatments, the data presented here suggest that CMV_TT_ exhibits favorable performance characteristics applicable to the development of therapeutic vaccines for allergies.

### Generation of a vaccine against Alzheimer’s disease

**Fig. 6:**
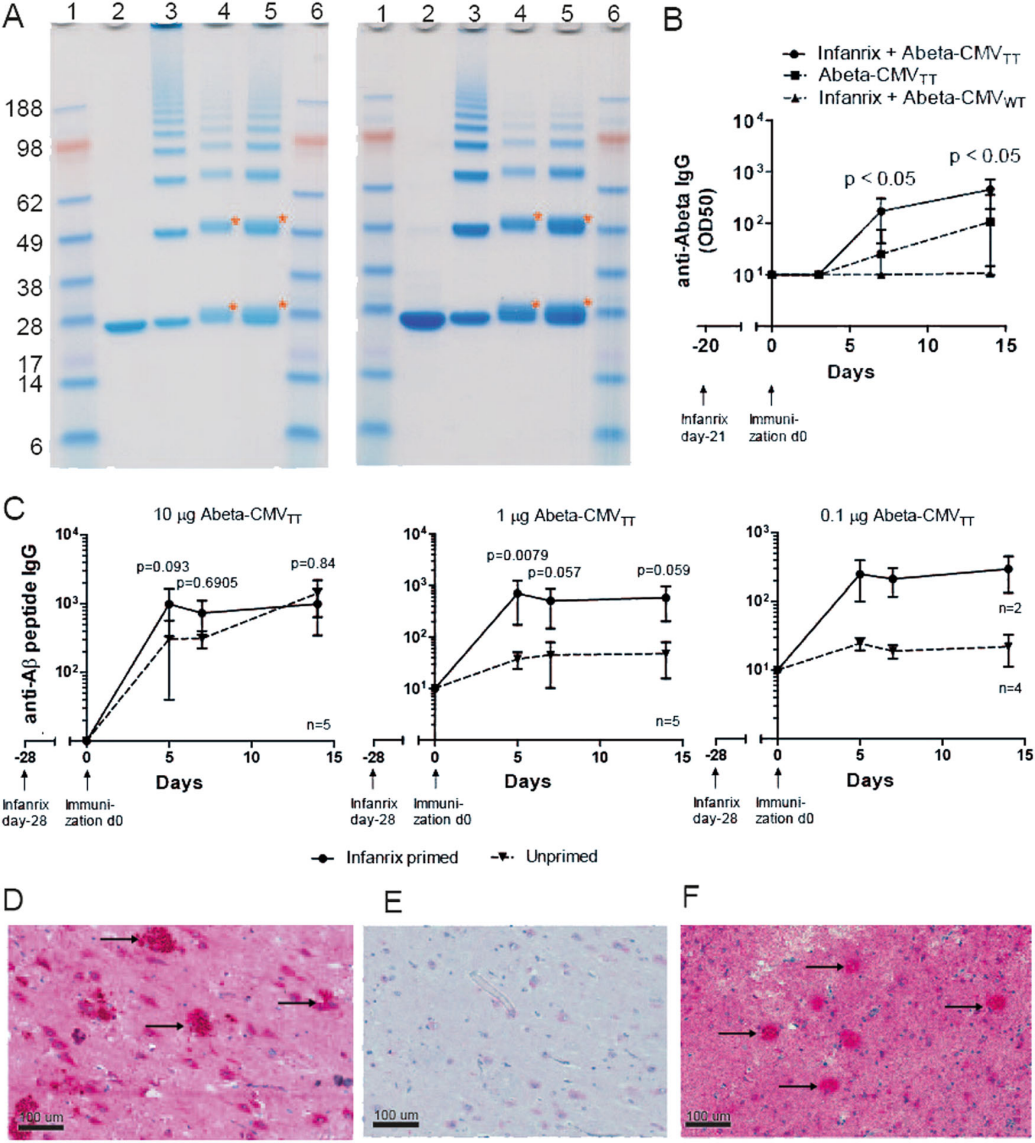
Immunogenicity of CMV_TT_ – based Alzheimer’s vaccine. **a** SDS-PAGE analysis (NuPage 4–12% Bis-Tris) of the Aβ_1–6_-CMV_TT_ (left panel) and Aβ_1–6_ -CMV WT (right panel) vaccine was run under denaturing and reducing conditions. Lanes from left to right: 1/6 - MW markers (Invitrogen, SeeBlue Prestained), 2-purified CMV VLP (~25 kDa coat protein monomer), 3-derivatized CMV-VLP after reaction with SMPH (bands in order of ascending size corresponding to VLP monomers, dimers, trimers etc. resulting from cross-linking), 4/5-Aβ-CMV following conjugation of the antigen amount loaded 5 µg (4) or 10 µg (5). Samples were derived from the same coupling experiment and were processed in parallel. **b** Immunogenicity assessment in young mice. One group of eight weeks old female C57BL/6 mice were primed with Infanrix and the other group was left untreated. After four weeks, mice were immunized with 10 µg, 1 µg or 0.1 µg of Aβ_1–6_ -CMV_TT_ vaccine. Blood was collected on day 0, 5, 7 and 14 and analyzed for Aβ-specific total IgG titers (endpoint). Mean values with SEM are shown of a representative experiment with *n* = 2–5 as indicated. Significances were obtained by a Mann Whitney test. **c** Immunogenicity assessment in old mice. Six months old Balb/C mice were primed with Infanrix® or left untreated. After 3 weeks, mice received 1 µg of Aβ_1–6_-CMV_TT_ or 1 µg of Aβ_1–6_–CMV WT vaccine intravenously. Blood was collected on day 0, 3, 7 and 14 and analyzed for Aβ-specific total IgG titers (OD50). Mean values with SEM are shown of a representative experiment with *n* = 8. Significances with *p* < 0.05 were obtained by a Mann Whitney test. **d–f** Immunohistochemistry of human brain section of the hippocampus showing the dentate gyrus with Cornu Ammonis of an Alzheimer’s patient. Formalin-fixed in paraffin embedded brain tissue of an Alzheimer’s patient was stained with **d** a mouse mAb generated against the Aβ peptide 1–17 as a positive control, **e** naïve mouse serum from day 0 as negative control and **f** purified serum from mice immunized with Aβ-peptide 1–6 (from N-terminus) coupled to CMV_TT_ VLP from day 56. All samples were counterstained with hematoxylin. Dimensions are depicted in pictures (100 µm). Arrows indicate single Aβ plaques

Alzheimer’s disease is one of the biggest issues in global health care. In terms of pathogenesis, treatments targeting both tau-protein (TauRx phase III), as well as β-amyloid^27^ have yielded disappointing results in clinical trials. Based on recent study results, it is possible that alternative and/or a combination of pathways need to be targeted. In addition, early intervention, preceding clinical symptoms, may be required. In terms of the so-called amyloid hypothesis, there is evidence that early dosing with antibodies specifically targeting N-terminal Aβ_1–42_ may be effective.^28^ Clearly, frequent administration of antibodies over decades in patients without clinical symptoms would be impractical. By contrast, a prophylactic vaccination approach could be a viable public health intervention. As most Alzheimer’s patients are elderly, the CMV_TT_ platform may confer significant advantages in terms of efficacy. In order to obtain initial data supporting this notion, we tested whether a CMV_TT_ based vaccine against Aβ_1–6_ would induce the species of antibodies thought to be protective, that is, IgG antibodies recognizing aggregated human Aβ_1–42_ in the brain of Alzheimer’s patients. To this end, we coupled Aβ_1–6_, i.e., the N-terminus of Aβ_1–42_ to CMV_TT_ (Aβ_1–6_-CMV_TT_) or VLPs without the tetanus epitope (Aβ_1–6_-CMV_WT_, Fig. 6a). The control vaccine as such is analogous to the CAD106 vaccine currently undergoing clinical testing (NCT02565511), as both are VLP-based vaccines coupled to the same epitope.

In order to show enhanced immunogenicity by vaccination with Abeta-CMV containing the TT epitope, 8 weeks old C57BL/6 mice were primed with Infanrix, a hexa-valent vaccine for children against Tetanus and other infections e.g., diphtheria, pertussis, hepatitis B, polio, and haemophilus influenza B. After four weeks, mice either primed with Infanrix or left untreated were immunized with 10 µg, 1 µg or 0.1 µg of Abeta-CMV TT vaccine and the anti-Abeta IgG response in serum was analyzed (Fig. 6b). In comparison to the unprimed control group, mice that were primed with Infanrix showed an increased immune response especially early on day 5 and 7 after vaccination against Abeta peptide independent of the dose amount.

In order to study whether the increased immunogenicity also holds true in older animals, six months old BALB/c mice were either pre-immunized against Tetanus or left untreated as control before immunization with 1 µg of Aβ_1–6_-CMV_TT_ or Aβ_1–6_-CMV_WT_. Vaccination with CMV_TT_ compared to CMV_WT_ vaccine already induced a stronger response regardless of whether mice had been primed against tetanus (Fig. 6c, compare “only Aβ -CMV_TT_” with “Infanrix + Aβ-CMV_WT_”). Immunogenicity of Aβ-CMV_TT_ vaccine was even further increased when mice were primed against tetanus (Fig. 6c, compare “Infanrix + Aβ-CMV_TT_” with “only Aβ-CMV_TT_”). Thus, young and old mice immune to tetanus mount an enhanced response to an Alzheimer’s vaccine based on CMV_TT_.

To assess whether the induced antibodies exhibited the right specificity, serum antibodies of mice immunized with Aβ_1–6_-CMV_TT_ vaccine were tested for their ability to recognize plaques of Aβ in brain sections from Alzheimer’s patients (Fig. 6d-f). Antibodies induced by active immunization with the Aβ _1–6_-CMV_TT_ vaccine (Fig. 6f) recognized Alzheimer’s plaques on human brain sections similar to as a monoclonal antibody raised against Aβ peptide 1–17 (Fig. 6d). Serum from immunized but not control mice recognized both Aβ_1–42_ by ELISA (not shown) as well as plaques in brain sections from Alzheimer’s patients, indicating that Aβ_1–6_-CMV_TT_ was able to induce antibody responses of the desired specificity (Fig. 6e,f).

## Methods

### Ethics statement

All studies involving either animal and/or human samples were subject to prior approval by the respective local ethics committees. For studies using mice, methods were performed in accordance with relevant regulations and guidelines. Methods were approved by the animal ethics research committee of the University of Dundee as part of the standard operating procedure for study approval for project licence 60/4944. Studies involving human tissue for Alzheimer histology were overseen by KEK Zürich ethics committee. Tissue was ascertained as part of a KEK-approved project (Adriano-Aguzzi/i2016). According to local governance regulation, use of anonymised slides for KEK-approved projecxts involving post mortem tissue is not subject to post-hoc individual consenting.

### Isolation and cloning of a coat protein (CP) of cucumber mosaic virus (CMV)

The total RNA from CMV-infected lily leaves collected from a private garden in Riga, Latvia, was isolated using TRI reagent (Sigma, Saint Louis, USA) in accordance with manufacturer’s recommendations. For cDNA synthesis, OneStep RT-PCR kit (Qiagen, Venlo, Netherlands) was used. For amplification of the CMV CP gene, the primer sequences were chosen following analysis of CMV sequences from GenBank: CMcpF (CACCATGGACAAATCTGAA TCAACCAGTGCTGGT) and CMcpR (CAAAGCTTATCAAACTGGGAGCA CCCCAGATGTGGGA); NcoI and HindIII sites underlined. The corresponding PCR products were cloned into the pTZ57R/T vector (Fermentas, Vilnius, Lithuania). *E. coli* XL1-Blue cells were used as a host for cloning and plasmid amplification. To avoid RT-PCR errors, several CP gene-containing pTZ57 plasmid clones were sequenced using a BigDye cycle sequencing kit and an ABI Prism 3100 Genetic analyzer (Applied Biosystems, Carlsbad, USA). After sequencing, the cDNA of CMV CP gene without sequence errors coding for CMV coat protein was then subcloned into the NcoI/HindIII sites of the pET28a(+) expression vector (Novagen, San Diego, USA), resulting in the expression plasmid pET-CMV_WT_.

To replace the original amino acids at the N-terminus of CMV CP against foreign epitope sequences, as a template for PCR amplification and mutagenesis the pET-CMV_WT_ plasmid was used. Internally in CMV_WT_ gene located SalI site (Fig.1) was used for cloning corresponding PCR products.

To introduce the tetanus toxoid epitope coding sequence in CMV_WT_ gene, two step PCR mutagenesis was necessary. For the first step amplification following primers were used for PCR: pET-220 (agcaccgccgccgcaaggaa –upstream from pET28a+ polylinker, the amplified region includes BglII site) and CMV-tt83-1R (ATTTGGAGTTGGCCTTAATATACT GGCCCATGGTATATCTCCTTCTTAAAGT). For the second round the PCR product from the first round was diluted 1:50 and re-amplified with primers pET-220 (SEQ ID NO: 11) and CMV-tt83Sal-R2 (GACGTCGACGCTCGGTAATCCCGATAAATTTGGAGTTG GCCTTAATATACTG).

To obtain CMV VLPs, *E. coli* C2566 cells (New England Biolabs, Ipswich, USA) were transformed with the CMV_TT_ CP gene-containing plasmid pET-CMV_TT_. After selection of clones with the highest expression levels of target protein, *E. coli* cultures were grown in 2xTY medium containing kanamycin (25 mg/l) on a rotary shaker (200 rev/min; Infors, Bottmingen, Switzerland) at 30 ^o^C to an OD600 of 0.8–1.0. Then, the expression was induced with 0.2 mM Isopropyl-β-D-thiogalactopyranoside (IPTG), and the medium was supplemented with 5 mM MgCl_2_. Incubation was continued on the rotary shaker at 20 ^o^C for 18 h. The resulting biomass was collected by low-speed centrifugation and was frozen at -20 ^o^C. After thawing on ice, the cells were suspended in the buffer containing 50 mM sodium citrate, 5 mM sodium borate, 5 mM EDTA, 5 mM mercapto-ethanol (pH 9.0, buffer A) and were disrupted by ultrasonic treatment. Insoluble proteins and cell debris were removed by centrifugation (13,000 rpm, 30 min at 5 ^o^C). The soluble CMV_TT_ CP protein in clarified lysate was pelleted using saturated ammonium sulfate (1:1, vol/vol) overnight at +4 ^o^C. Soluble CMV_TT_ CP-containing protein solution was separated from the cellular proteins by ultracentrifugation (SW28 rotor, Beckman, Palo Alto, USA; at 25,000 rpm, 6 h, 5 ^o^C) in a sucrose gradient (20–60% sucrose in buffer A, without mercapto-ethanol, supplemented with 0.5% Triton X-100). After dialysis of CMV-containing gradient fractions, VLPs were concentrated using ultracentrifuge (TLA100.3 rotor, Beckman, Palo Alto, US; at 72,000 rpm 1 h, +5 ^o^C) or by ultrafiltration using Amicon Ultra 15 (100 kDa; Merck Millipore, Cork, Ireland). All steps involved in the expression and purification of VLP were monitored by SDS-PAGE using 12.5% gels.

### Characterization of CMV_TT_ VLPs

The concentration of purified CMV_TT_ was estimated using the QuBit fluorometer in accordance with manufacturer’s recommendations (Invitrogen, Eugene, USA). Concentrated VLP solutions (approximately 3 mg/ml) were stored at+ 4 ^o^C in 5 mM sodium borate, 2 mM EDTA, buffer (pH 9.0). To demonstrate the presence of tetanus epitope in CMV VLPs, the mass spectrometric analysis of the purified CMV_TT_ (also referred to as CMV-Ntt830) VLPs was used. The stability of VLPs was investigated by thermal denaturation in the presence of SYPRO-orange dye using a DNA melting point determination program and a real-time PCR system MJ Mini (Bio-Rad, Hercules, USA). This involved subjecting VLP samples to increasing heat whilst monitoring fluorescence. The morphology of VLPs was confirmed by applying samples (0.1–0.5 mg/mL) on glow discharged carbon coated copper grids and observing by transmission electron microscopy using JEM-1230 electron microscope (JEOL, Tokyo, Japan). Average sizes of VLP preparations were determined by dynamic light scattering, assessed by diluting to 1 mg/ml and analyzing in Zetasizer Nano ZS instrument (Malvern Instruments Ltd, Malvern, UK).

### Trypsin-fingerprint/mass spectrometry

Reactive Lys residues available for chemical coupling of antigens were determined by FITC-labeling free amino groups, FITC-modified coat protein was separated in SDS/PAGE gel, the fragment was excised from gel and digested with trypsin (Trypsin Profile IGD Kit, Sigma). VLPs (1.5 mg/ml in 50 mM Sodium borate, 2 mM EDTA, pH 9.0) were reacted with 20 mM FITC at +4 ^o^C for 24 h. Samples after FITC treatment were separated in 12% SDS-PAGE, protein spots after Coomassie staining excized from gel and treated with Trypsin using Trypsin Profile IGD Kit and protocol (Sigma, St Louis, USA) ON +4 ^o^C. The reaction mixture was purified using ZipTip tips (Merck Millipore, Cork, Ireland), diluted with a 3-hydroxypicolinic acid matrix solution and spotted onto an MTP AnchorChip 400/384TF. MALDI-TOF MS analysis was carried out on an Autoflex MS (Bruker Daltonik, Bremen, Germany). The protein molecular mass (MM) calibration standard I (3–20 kDa; Bruker Daltonik) and Peptide calibration standard II was used for mass determination.

### T cell proliferation assays

Blood was obtained from human volunteers and peripheral blood mononuclear cells (PBMC) were isolated by density gradient centrifugation on Ficoll Paque (GE Healthcare, Chalfont St. Giles, UK). PBMCs were labeled with 10 µM CFSE (Biolegend, San Diego, CA, USA) according to the manufacturer’s protocol and cultured in RPMI-1640 (Gibco, Basel, Switzerland) supplemented with 5% of heat-inactivated human AB serum (Swiss Red Cross, Bern, Switzerland), 2 mM L-Glutamine (Biochrom, Berlin, Germany), 50 U/ml penicillin and 50 µg/ml streptomycin (Bioconcept, Allschwil, Switzerland). During 7 days, the cells were stimulated with either 1 µg/ml CMV WT or CMVTT or left untreated. T cells were then stained with anti-CD3-PerCp-Cy5.5, anti-CD4-PE-Cy7 (Biolegend, San Diego, CA, USA), data acquired with BD FACSCanto™ and analyzed with FACS-Diva software (BD Biosciences, Franklin Lakes, NJ, USA). This study was approved by the local ethics committee.

### CryoEM reconstruction to determine CMV_TT_ VLP structure

CMV_TT_ (2.5 mg/ mL) in 5 mM sodium borate, 2 mM EDTA, pH 9 was applied for 20 s to glow-discharged holey carbon-coated copper grids (C-flat, CF-2/1–2 C; Protochips) in a humidified chamber (70% relative humidity), excess sample being removed by blotting on to filter paper and grids rapidly vitrified by plunging in to liquid ethane (Vitrobot, FEI). Cryo-grids were stored in liquid nitrogen prior to imaging with a Tecnai F30 Polara microscope (FEI).

CryoEM images were collected using a Tecnai F30 ‘Polara’ microscope (FEI) at 300 kV, equipped with an energy filter (GIF Quantum, Gatan) operating in zero-loss mode (0–20 eV energy selecting slit) and a direct electron detector (K2 Summit, Gatan). Movies (25 frames, each 0.2 s) were recorded at 1.0–3.0 μm underfocus in single-electron counting mode with SerialEM at a calibrated magnification of 37,037×, thus resulting in a pixel size of 1.35 Å. Frames from each movie were aligned and averaged to produce drift-corrected micrographs.^27^ Data are summarized in Table S1.

Structures were solved with RELION 1.3 according to recommended gold-standard refinement procedures,^28^ and icosahedral symmetry was applied. Micrographs showing signs of astigmatism or significant drift were discarded and not used for further analysis. Reference-free 2D class averaging was used to discard distorted particles. The particle population was further improved by 3D classification. The X-ray structure of native CMV (PDB: 1F15)^29^ was low-pass-filtered to 50 Å and used as an initial template for 3D classification and refinement. A total of 3582 CMVtt830 particles from 6600 micrographs were used to solve the final density maps at 4.2 Å resolution, as indicated by Fourier shell correlation and 0.143 cut-off.

Using a similar approach as previously described,^30,31^ the CMV was fitted in the density map as a rigid body with UCSF Chimera 32. The fitting was improved further by real-space refinement using COOT 33 and Phenix 35. Only the coordinates being refined each time, with the maps kept constant. Cross-correlation guided each round of model optimization between the map and the model.

### Production of mIL17 protein

A nucleotide fragment codon optimized for *E*. *coli* was synthesized (GeneArt Genestring, ThermoFisher Scientific, UK) encoding the murine IL-17 (aa 26–158, accession: NP_034682) preceded by sequences for an initiating methionine and glycine and coding region for a hexahistidine tag at the N-terminus of the encoded protein, was followed by additional sequences to give a polylinker of 5 glycine and terminal cysteine at the C- terminus. This fragment was cloned by restriction digest (NcoI/ BamHI, NEB, UK) in to a similarly double digested prokaryotic expression vector (pET28b, Merck, UK). Resulting clones were confirmed by PCR colony screen and sequenced using T7 promoter forward primer (TAATACGACTCACTATAGGG) and pET-RP reverse primer (CTAGTTATTGCTCAGCGG).

Murine IL-17 was expressed in *E. coli* strain BL21(DE3)Star (Thermo Fisher Scientific, UK), culturing to OD600 of 0.7 and inducing with 1 mM IPTG overnight (16–18 h) at 37 ^o^C. Harvested cells were lysed in PBS, pH 7.4 supplemented with Lysonase™ as per manufacturers recommendation (Merck, UK), incubating 1 h and sonicating lysate on ice (3 cycles of 20 s on, 20 s off, 40% power amplitude with a VibraCell fitted with microtip, Sonics and Materials Inc, US). The lysate was clarified by high speed centrifugation (15,000×*g*, 30 min) and the pellet was retained. The recombinant IL-17 was purified from the insoluble fraction as previously described.^18^ Briefly, inclusion bodies (IBs) from the insoluble fraction were subjected to sequential washes with 50 mM Tris pH 8, 10 mM EDTA, 100 mM NaCl buffer (initially containing 0.5% Triton × 100 then 2% Triton × 100, then without detergent). The IBs were solubilized with solubilization buffer (6 M guanidine HCl, 10 mM Tris.HCl, 100 mM NaH_2_PO_4_, 2 mM beta-mercaptoethanol (BME), pH 8), insoluble debris removed by centrifugation at 10,000 *g*, 20 min and clarified soluble fraction subjected to nickel affinity chromatography (HisTrap Excel, GE Healthcare) under denaturing conditions with low pH elution (50 mM NaH_2_PO_4_, 100 mM Tris, 6 M Gdn•HCl, 2 mM BME with Buffer A, adjusted to pH 6.15; and Buffer B adjusted to pH 4). This was followed by concentration (Amicon Ultra 3000 centrifugal filters, Merck, UK) and further purification using size exclusion chromatography (HiLoad Sephadex 75, GE Healthcare, UK).

Fractions containing purified mIL-17 as identified by a 16 kDa band (predicted molecular mass of 16,378 Da) by Coomassie stained SDS-PAGE were pooled. Note that proteins were ethanol precipitated and resuspended in reducing Laemmli buffer to remove guanidine prior to the analysis by SDS-gel electrophoresis. The purified mIL-17 was still denatured and therefore required refolding to native form. Sample was adjusted to a concentration of < 0.2 mg/mL and refolding followed a 2-step dialysis strategy. Dialysis tubing was subject to 3 buffer exchanges (2 L each) over 24 h with 0.5 M Arginine, 50 mM sodium phosphate (pH 8), 10% (v/v) glycerol and redox pair (5 mM reduced glutathione, 0.5 mM oxidized glutathione). For the second step of dialysis, samples were dialyzed for a further 24 h in to 50 mM sodium phosphate (pH 8), 10% (v/v) glycerol, with at least 2 buffer exchanges (2 L each).

### Production of psoriasis vaccine

The purified and refolded mIL-17 was conjugated to CMV_TT_ via the heterobifunctional crosslinker, Succinimidyl-6-[(beta-maleimidopropionamido)hexanoate] (SMPH, Thermo Fisher Scientific, UK). VLPs were reacted with 7.5-molar excess of SMPH, for 30 min at room temperature (RT), unreacted excess crosslinker being removed by 3 repeated diafiltration steps using 100 k centrifugal filters. The recovered derivatised VLPs were then mixed at equimolar concentration with mIL17 that was pre-treated 60 min at RT with 10-molar excess TCEP to liberate C-terminal cystines (dimeric IL-17 has intramolecular disulfide bonds forming a cysteine knot that are typically resistant to reducing reagents without denaturation). Coupling efficiency was estimated by densitometry analysis of CMV_TT_ bands in Coomassie stained SDS-PAGE, comparing proportions of monomer net intensity (NI) and monomer plus IL-17 NI after correcting for relative MW (See figure S3B). Densitometric values were provided using Image Lab 5.0 software (Bio-rad Laboratories) or equivalent image analysis software. Coupling or epitope density for IL17 per VLP was calculated as follows (eq1): IL17-CMV(NI/MW) / (Σ VLP(NI/MW) + IL17-CMV(NI/MW)) x number of protein per subunit = epitope density (ED). Expressed as a percentage this gives an estimated coupling density of IL17-CMV of 8%, meaning a minimum of 14 molecules of IL17 were covalently linked to each particle (consisting of 180 CMV monomers).

### In vivo clinical efficacy and immunogenicity of the IL17 vaccine in mice

In vivo clinical efficacy was measured by dorsal skin and ear epidermal thickness assessed macroscopically and with H&E histology. Ear thickness measurements and body weight measurements were recorded at different time intervals during the experiments using a micrometer (Mituyo catalog nr 7301, Thickness Gage Series 7 Flat Anvil Type Dial) and a scale, respectively. Ear samples and dorsal skin samples were taken, roughly 2 cm by 2 cm, and fixed in neutral buffered 10% formalin. 2 ml of formalin was used per 100 mg of tissue. Tissues were fixed for a minimum of 48 h at RT. Tissue was then dehydrated through a series of graded ethanol immersions. Once fixed, the tissue was processed as follows on a Leica Peloris tissue processor: formalin × 2, 95% ethanol × 4, 99% ethanol × 4, xylene, 99% ethanol first paraffin wax and second paraffin wax. Wax was melted by placing tissue cassettes in 58 ^o^C paraffin bath for 15 min. Mold that left 2 mm margin of paraffin wax around the tissue was selected. Molten paraffin was dispensed in mold and warm forceps were used to transfer tissue into the mold, placing the cut side down. The mold was transferred to a cold plate and gently the tissue was pressed flat. With the tissue in the desired orientation the labeled tissue cassette was added on top of the mold as a backing. After 30 min the wax was cooled and hardened and the paraffin block is popped out of the mold. Tissues were then sectioned using a Leica microtome to 4 µM thickness. The sections were dried in an oven at 37 ^o^C. The tissue and paraffin attached to the cassette has formed a block, which was stored at RT. Haematoxylin and Eosin staining was performed using Leica Autostainer XL. The procedure was as follows: tissue was placed in an oven at 60 ^o^C for 15 min. Deparaffinise sections, 3 changes of xylene, 30 s each. Re-hydrate in 2 changes of absolute alcohol, 30 s each. 99% alcohol for 2 min and 95% alcohol for 2 min. Wash in water for 30 s. Stain in Harris haematoxylin solution for 4 min. Wash in running tap water for 1 min. Differentiate in 0.1% acid alcohol for 1 min. Wash in water for 1 min. Bluing in saturated lithium carbonate solution for 1 min. Counterstain in eosin solution for 20 s. Wash in tap water for 30 s. Rinse in 95% alcohol for 30 s × 2 and rinse in 99% alcohol for 30 s × 2. Rinse in Isopropyl alcohol for 30 s. Clear in 3 changes of xylene, 30 s each. Sections were coverslipped by Leica Coverslipper CV5030 using DPX mountant.

Immunogenicity was assessed via measuring anti-IL-17A serum levels with a series of ELISA experiments. Serum samples were acquired at baseline, at each vaccination point and 2 weeks post final vaccination. Samples of up to 200 µl were acquired at each point with tail vein blood sampling. First Nunc-Immuno 96 clear polystyrene MicroWell solid plates were coated as follows. Recombinant murine (rm) IL-17 was diluted to a concentration of 2 µg/ml in PBS and vortexed to allow adequate mixing. 100 µl of this mixture was added to each well of the plate using a multi-channel pipette. The plate was covered with parafilm and stored at 4 ^o^C for up to 72 h. Wash buffer was made using PBS with 0.05% Tween20 and blocking buffer made from the washing buffer composed of 2% BSA in PBS with 0.05% Tween20. The plate was removed from the fridge, the liquid expelled and the plates washed 3 times with 200 µl wash buffer per well. 250 µl blocking buffer was added to each well and the plates left for 150 min. Serum was serial diluted in wash buffer on 96-well plates to the concentrations 1:10, 1:100 etc. Control serum, taken before any antibody or vaccination injections, was used in each experiment and diluted to a concentration of 1:100. This high concentration relative to the test samples was used to allow for enough serum to be used for all the experiments. Liquid was discarded from the blocked plate and the plate was washed 3 times as above. 50 µl of diluted serum was transferred onto the coated plate, with each sample a duplicate of that sample was used. Empty wells were filled with 50 µl sterile water for standardization purposes and the plate incubated for 90 min on the shaker. Detection antibody was made using Anti-mouse IgG labeled to alkaline phosphatase diluted in blocking buffer to a concentration of 1:5000. Liquid was discarded from the blocked plate and the plate was washed 3 times as above. 100 µl of detection antibody was added to each well and the plates covered with parafilm and left to incubate on the shaker for 45 min. Liquid was discarded from the blocked plate and the plate was washed 3 times as above. 50 µl of alkaline phosphatase yellow (pNPP) liquid substrate was added to each well and the wells incubated on the shaker for 20 min. 5 µl of stop solution (3 M NaOH) was added to each well and the plate read in the SpectraMaxM3 spectrophotometer at 405 nm. SoftMax Pro6.3 software was used to read the plates. Absorbance instruments with endpoint settings of 405 nm wavelength were used. ELISA data were analyzed using a 3-parameter logistic fit according to standard sigmoid curve equation, *Y* = MAX/(1 + (x/EC50)^(SLOPE)) where the minimum was set to zero after subtracting the H20 optical densities from test values. Curve fitting was done using the ‘solver’ add-in using the GRC-nonlinear fitting algorithm.

Residual tail vein blood samples known to be highly immunogenic for IL-17A were used to test the specificity of the immune response generated by the vaccine on ELISA plates coated with IL-17A and IL-17F. The same blood samples were tested for both IL-17A and IL-17F and the ELISA was run for both at the same time to minimize variables.

### Expression and purification of Fel d 1

The sequence of Fel d 1 antigen ^25,29,30^ comprises of chain 1 and chain 2 that were genetically fused via a 15-aa linker sequence (GGGGS)_3_ and synthesized by GeneArt (Thermo Fisher Scientific, Germany). The COOH-terminus was modified by a histidine tag (6x His) for purification purposes followed by a small spacer (GGCG) including a cysteine for coupling. Our version of the recombinant Fel d 1 has a predicted molecular mass of 20,147 Da. The encoding Fel d 1 sequence was cloned into the high level-expression plasmid vector pET42. *E. coli* C2566I (NEB) were transformed with the plasmid and cultivated at 37 °C. At a OD600 between 0.5 and 0.7 1 mM IPTG was added to the culture and placed at 20 °C overnight for soluble protein expression. Cells were harvested and separated from supernatant by centrifugation at 4800×*g* for 15 min at 4 °C. The cell pellet was resuspended in 50 mM NaH_2_PO_4_, 300 mM NaCl, 10 mM imidazole, pH 8.0 and sonicated for cell lysis. The solution containing the recombinant Fel d 1 was cleared from cell debris by centrifugation at 16,000×*g* for 30 min at 4 °C. The supernatant was collected and purified by a Ni-NTA (Qiagen) column using the ÄKTA purifier. Recombinant Fel d1 containing a His-tag bound to Ni-NTA and was washed with 50 mM NaH_2_PO_4_, 300 mM NaCl, 50 mM imidazole, pH 8.0, finally eluted in 50 mM NaH_2_PO_4_, 300 mM NaCl, 250 mM imidazole, pH 8.0 and dialyzed against PBS overnight at 4 °C. The antigen authenticity was tested in a capture ELISA (EL-FD1, Indoor Biotechnology) using mAbs that were raised against natural Fel d 1.

### Production and immunogenicity testing of Fel d 1 vaccine

The purified Fel d 1 was conjugated to CMV_TT_ VLPs using the crosslinker SMPH. A 10-molar excess of SMPH reacted at 23 °C for 60 min shaking at 350 rpm. The side product was removed using PD10 desalting columns (GE Healthcare). To gain access to the cysteine at the COOH-terminus of the recombinant Fel d 1, the antigen solution reacted with a 10-molar access of a mild reducing agent TCEP (Sigma) before a 2-molar excess of Fel d 1 was added to the derivatized CMV_TT_-VLP at 23 °C for 3 h at 350 rpm. Unbound Fel d 1 protein was removed by Amicon Ultra centrifugal filter devices (Millipore) and tested by SDS-PAGE analysis. CMV monomers appear at ~25 kDa and Fel d 1 protein at ~20 kDa. Due to crosslinking of subunits, derivatization by SMPH leads to the characteristic ladder of CMV monomers, dimer, trimers, tetramers, etc. The primary coupling band appears as one CMV monomer linked to one Fel d 1 protein at ~45 kDA. Coupling efficiency was calculated by densitometry (as previously described for IL17A-CMV_TT_ vaccine) with a result of ~ 30% meaning 60 Fel d 1 molecules were linked to one particle.

To test immunogenicity, 8 weeks old Balb/c were immunized with 1 µg of free Fel d 1, Fel d 1 mixed with or coupled to CMV_TT_ VLPs in 150 µl PBS intravenously. Blood was collected at different time points and analyzed for anti-Fel d1 antibodies by ELISA. Natural Fel d 1 (Indoor Biotechnologies) at 1 µg/ml was applied overnight to NUNC ELISA plates which were then washed and blocked with 2% BSA in PBS Tween 20 (0.05%). After washing, serially diluted mouse sera were applied to the plates. After further washing, goat anti-mouse IgG antibody labeled with horseradish peroxidase was applied to the plates. Following a final washing step, O-phenylenediamine dihydrochloride was added and, after 7 min, the reaction stopped with 5% sulfuric acid. The conversion of OPD by HRPO was measured at 450 nm. The titers is reported as OD50 which is the reciprocal of the dilution which reaches half of the maximal OD value.

### Basophil activation test (BAT)

The BAT assay (Bühlmann Co.) using whole blood from allergic patients was tested for the up-regulation of the degranulation marker CD63 on basophils (CCR3+)^31^ upon incubation with Fel d 1 and Fel d 1 couples to CMV_TT_-VLPs by flow cytometry using a BD Fortessa. Briefly, 100 μl of stimulation buffer was mixed with 50 μl of EDTA-treated whole blood. In addition, 50 μl of dilutions of natural Fel d1, recombinant Fel d1 and recombinant Fel d 1 conjugate (Fel-CMV_TT_) were added. Positive control solutions including an mAb against FcεRI as well as an unspecific cell activator (fMLP) were also tested in the assay. Staining dye (20 μl per sample), containing anti-CCR3 Ab labeled to PE and anti-CD63 Ab labeled to FITC, was added and incubated at 37 °C for 25 min. Erythrocytes were subsequently lysed adding lysis buffer. After 10 min incubation, the samples were centrifuged at 500×*g* for 5 min and washed with wash buffer (PBS containing 2% FCS). After a second centrifugation step, the cell pellets were resuspended in wash buffer and acquired using a flow cytometer (FACS BD Fortessa). The samples were analyzed by DIVA software. The percentage of the CD63 expression on CCR3+ basophils was analyzed.

### Fel d 1 allergen challenge *in-vivo*

For Fel d 1 sensitization, two groups of 5 female 6-wk-old BALB/c mice were injected with 1 μg natural Fel d 1 (Indoor biotechnologies) mixed in 200 µl Alum (10 mg/ml Al(OH)3; Alhydrogel adjuvant 2%, InvivoGen) at day 0, 7 and 14 intraperitoneal. At day 28 mice were vaccinated with 30 μg vaccine Fel-CMV_TT_ or only CMV_TT_-VLPs subcutaneously.

For the induction of anaphylaxis, two weeks after vaccination on study day 42 mice were challenged with 3 μg natural Fel d1 in 150 μl PBS intravenously. The rectal core temperature was measured with a rectal probe digital thermometer (MiniTemp, Vetronic Services LTD) every 10 min for 50 min after intravenous antigen challenge.

### Production of Alzheimer’s vaccine

CMV_TT_ VLPs or CMV wildtype (WT) VLPs reacted with a 7.5-molar excess of the crosslinker SMPH for 1 h at RT. A short 6-aa sequence (DAEFRH) from the N-terminus from the full length Aβ peptide (1–42 aa) encoding with terminal cysteine residue (GGC) purchased from Dr. Petra Henklein, Charite, Berlin, Germany was conjugated in a 5-molar excess to the derivatised CMV_TT_ or CMV_WT_ particles for 3 h at RT. The vaccine was analyzed by SDS-PAGE to confirm coupling of peptide to CMV_TT_ or CMV_WT_ particles. CMV monomers appear at ~25 kDa. Due to crosslinking of subunits, derivatization by SMPH leads to the characteristic ladder of CMV monomers, dimer, trimers, tetramers, etc. Coupling bands appear above CMV monomer when one peptide (~1 kDa) bound to CMV momoner (coupling product ~26 kDa) or two peptides bound to CMV monomer (coupling product ~27 kDa). Coupling bands of peptide(s) to CMV dimers appear above CMV dimer accordingly. The coupling efficiency (described previously for IL17A-CMV_TT_ vaccine) was calculated by densitometry resulting in ~60% meaning approximately 108 peptide molecules were linked to each VLP.

### Immunogenicity assessment of Alzheimer’s vaccine and assessment of TT enhancement

To test the TT enhancement, 6 months old female Balb/C mice were primed with Infanrix (1/5 dose per mouse). A control group of mice were left un-primed. After three weeks, mice were immunized with 1 µg Aβ_1–6_-CMV_TT_- or Aβ_1–6_-CMV_WT_- vaccine intravenously. Blood was collected on days 0, 3, 7 and 14 and analyzed for serum Aβ specific IgG antibodies as described previously. The antibody titers is reported as OD50 which is the reciprocal of the dilution which reaches half of the maximal OD value.

### Immunohistology of Alzheimer’s patients’ brain sections

Sections of paraffin embedded brain (hippocampus) tissue of an Alzheimer’s patient were prepared with a microtome. After deparaffinising, sections were blocked in BSA with normal horse serum. A monoclonal antibody raised against Aβ_1–17_ (Abcam ab11132), or by Protein G-purified mouse serum from day 0 (pre-immune) or day 56 (post-immune) after one immunization were applied to sections for 1 h at RT. To detect specific binding all pre-treated brain sections were incubated with a biotinylated horse anti-mouse IgG (Vectorlabs BA2000) for 30 min at RT, first and next, incubated with streptavidin labeled with AP. Binding was visualized by substrate Fast Red from Sigma. Subsequently, in order to visualize nuclei a counterstain using hematoxylin was performed. Stained slides were digitalized at 0.25 μm per pixel resolution using a ScanScope XT (Aperio, Vista, CA, USA). Full-slide scans were stored as high-resolution (0.21 microns/pixel). Regions of interest were processed using ImageScope software (Aperio).

### Data availabality statement

The cryo-electron microscopy density map for CMV-tt VLP structures have deposited with the Electron Microscopy Data Bank: EMD-3855 and associated atomic coordinates submitted to the Protein Data Bank with accession number PDB: 5OW6.

## Electronic supplementary material

Supplement

Original gel files Fig1c

Original gel file Fig1D

Full length gel blot Fig 5A

Full length gel blot Fig 6A_left panel

Full length gel blot Fig 6A_right panel

Mol weight marker for Figure 5A, 6A

Original gel file FigS3a

Original gel file FigS6
