# Supplementary material for: Incorporation of tetanus-epitope into virus-like particles achieves vaccine responses even in older recipients in models of psoriasis, Alzheimer’s and cat allergy
Source: NPJ Vaccines. 2017 Oct 23;2:30. doi: 10.1038/s41541-017-0030-8 (PMC5653761; doi:10.1038/s41541-017-0030-8)
Supplement: Supplementary file 1 — Supplement [file 41541_2017_30_MOESM1_ESM.pdf]

Supporting Material to: "Incorporation of Tetanus-epitope into virus-like particles achieves vaccine responses even in older recipients in models of psoriasis, Alzheimer's and cat allergy

SUPPLEMENTARY METHODS 2

1. Cloning, expression and VLP isolation of plant VLPs 2

2. Construction of T-cell epitope containing plant VLPs 3

SUPPLEMENTARY TABLE S1. 5

SUPPORTING FIGURES. 6

Supporting Figure S1 6

Supporting figure S2 7

Supporting Figure S3. 8

Supporting Figure S4 9

Supporting figure S5 10

Supporting figure S6 11

Supplementary Methods

1. Cloning, expression and VLP isolation of plant VLPs

A. Cocksfoot mottle virus (CfMV) VLPs. The viral RNA from purified CfMV particles* was isolated using TRI reagent (Sigma, Saint Louis, USA). For cDNA synthesis, M-MuLV H (-) reverse transcriptase (Fermentas, Vilnius, Lithuania) and random hexamer primers were used. To amplify the CP gene, following oligonucleotides were used for PCR reaction: CfcpF (ataccatggtggtgaggaaaggagcagca) and CfcpR (TCTAAGCTTCTACAAATTTGTAGAAGGGGAAACTGATC). The corresponding PCR products were cloned into the pTZ57R/T vector (Fermentas, Vilnius, Lithuania). E. coli XL1-Blue cells were used as a host for cloning and plasmid amplification. To avoid RT-PCR errors, several CfMV CP gene-containing pTZ57 plasmid clones were sequenced using a BigDye cycle sequencing kit and an ABI Prism 3100 Genetic analyzer (Applied Biosystems, Carlsbad, USA). After sequencing, the cDNA of CfMV CP gene without sequence errors coding for CfMV coat protein was then subcloned into the NcoI/HindIII sites of the pET28a(+) expression vector (Novagen, San Diego, USA), resulting in the expression plasmid pET-CfMV. To obtain CfMV VLPs, E. coli C2566 cells (New England Biolabs, Ipswich, USA) were transformed with plasmids pET-CfMV and pACYC-RIL (Stratagene, USA; codes for rare E.coli amino acid codon tRNA genes - Arg, Ile, Leu). Obtained E. coli culture was grown in 2xTY medium containing kanamycin and chloramphenicol (25 mg/l) on a rotary shaker (200 rev/min; Infors, Bottmingen, Switzerland) at 30oC to an OD600 of 0.8–1.0. Then, the cells were induced with 0.2 mM IPTG, and the medium was supplemented with 2 mM CaCl2 5 mM MgCl2. Incubation was continued on the rotary shaker at 20oC for 18 h. The resulting biomass was collected by low-speed centrifugation and was frozen at -20oC. After thawing on ice, the cells were suspended in the buffer containing 15 mM KH2PO4 pH 5.5, 5 mM β-mercaptoethanol, 1 mM PMSF, 0.5 M urea (buffer A) and were disrupted by ultrasonic treatment. Insoluble proteins and cell debris were removed by centrifugation (13,000 rpm, 30 min at 5oC). The soluble CfMV CP protein in clarified lysate was pelleted using 8% PEG8000 and 1.0 M NaCl overnight at +4oC. CP-containing protein solution was separated from the cellular proteins by ultracentrifugation (SW28 rotor, Beckman, Palo

Alto, USA; at 25,000 rpm, 6 h, 5oC) in a sucrose gradient (20–60% sucrose in buffer A, without mercaptoethanol and urea, supplemented with 0.5% Triton X-100). After dialysis of CMV-containing gradient fractions, VLPs were concentrated using ultracentrifuge (TLA100.3 rotor, Beckman, Palo Alto, US; 72,000 rpm 1 h, +5oC).

*- Tars et al., Virology (2003) 310: 287-297

B. Raspberry bushy dwarf (RBDV) VLPs. RBDV-infected raspberry leaves were obtained from Dr. Sarmite Strautina (Dobele, Latvia). After isolation of total RNA with TRI reagent the cDNA was synthesized, using reverse transcriptase and random hexamer primers. For PCR, following oligonucleotides were used : RBcpF (ataccatggtctgcaagtactgtaatcaca) and RBcpR (TCTAAGCTTTCACAATTCTAACAAATCTTCGTCGA). The PCR product was cloned into the pTZ57R/T vector. RBDV CP gene without sequence errors was further subcloned into the NcoI/HindIII sites of the pET28a(+) expression vector, resulting in the expression plasmid pET-RBDV. All procedures, including the usage of helper plasmid pACYC-RIL for RBDV CP synthesis in E.coli C2566 and VLP purification were similar to that of CfMV VLPs.

2. Construction of T-cell epitope containing plant VLPs

A. Tetanus toxoid epitope-containing CfMV (CfMVTT) VLPs. To add TT-epitope QYIKANSKFIGITE at C-terminus of the CfMV CP (CfMVTTC) or insert between original amino acids (CfMVTTIN) , two primer pairs were used in PCR reactions, using pET-CfMV as a template: Cf-SauF (agcactcctgaggaatcggag)/Cf-ttC R(TCTAAGCTTACTCGGTAATCCCGATAAATTTGGAGTTGGCCTTAATATACTGGCCGGACAAATTTGTAGAAGGGGA) and Cf-ttIN-F (ACTCCTGAGGGATCCGGCCAGTATATTAAGGCCAACTCCAAATTTATCGGGATTACCGAATCGGAGAACGCTCATTTAACTG) / CfcpR. Resulting PCR products were subcloned in SauI/HindIII site of pET-CfMV. After identification of correct clones by sequencing the expression vectors pET-CfMVTTC and pET-CfMVTTIN were used for E. coli C2566 cell transformation. As previously described, also here the cotransformation with helper plasmid pACYC-RIL was necessary.

All further procedures, including the conditions for cultivation and VLP purification were similar to that of pET-CfMV. Purified CfMV-derived proteins were analyzed under EM.

The resulting sequence of CfMVTTC is following:
mvvrkgaatkapqqpkpkaqqqpggrrrrrgrsmepvsrplnppaavgstlkagrgrtagvsdwfdtgmitsylggfqrtagttdsqv
fivspaaldrvgtiakayalwrpkhweivylprcstqtdgsiemgflldyadsvptntrtmasstsfttsnvwgggdgssllhtsvksmgn
avtsalpcdefsnkwfklswstpeesenahltdtyvparfvvrsdfpvvtadqpghlwlrsrillkgsvspstnlsgqyikanskfigite;
CfMVTTIN sequence:
mvvrkgaatkapqqpkpkaqqqpggrrrrrgrsmepvsrplnppaavgstlkagrgrtagvsdwfdtgmitsylggfqrtagttdsqv
fivspaaldrvgtiakayalwrpkhweivylprcstqtdgsiemgflldyadsvptntrtmasstsfttsnvwgggdgssllhtsvksmgn
avtsalpcdefsnkwfklswstpegsgqyikanskfigitesenahltdtyvparfvvrsdfpvvtadqpghlwlrsrillkgsvspstnl

B. Tetanus toxoid epitope-containing RBDV (RBDVTT) VLPs. To replace the original amino acids of RBDV CP (SQKKLDASFVGFSE) against tetanus toxoid epitope (qyikanskfigite), the following primers were used for PCR: RBcpF and TT-SacR (TAGAGCTCTCGGTAATCCCGATAAATTTGGAGTTGGCCTTAATATACTGGCCGGAAACCTTG ATGGCT, SacI site underlined); resulting PCR product was subcloned in NcoI/SacI sites of pET-RBDV. The correct clone was identified by sequencing and designated as pET-RBDVTT. All procedures, including the construction of expression strain and VLP purification were similar to that of pET-CfMV. . Purified VLPs were analyzed using EM. The resulting sequence of RBDVTT is following:

MAKKAVPPIVKAQYELYNRKLNRAIKVSGQYIKANSKFIGITESSNPETGKPHADMSMSAKVKRV
NTWLKNFDREYWDNQFASKPVPRPAKQVLKGSSSKSQQRDEGEVVFTRKDSQKSVRTVSYWVCT
PEKSMKPLKYKEDENVVEVTFNDLTAQKAGDKLVSILLEINVVGGAVDDKGRVAVLEKDAAVTV
DYLLGSPYEAINLVSGLNKINFRSMTDVVDSIPSLLNERKVCVFQNDDSSSFYIRKWANFLQEVSAV
LPVGTGKSSTIVLT

Supplementary Table S1.

CryoEM data collection and refinement statistics

**CryoEM Detector**	**K2 Summit**
**Data set**	**CMV_{TT}**
Movies	353
Frames	25
Particles	3582
Pixel Size (Ǎ)	1.35
Defocus Range (μm)	1.5-3.0
Voltage (kV)	300
Electron Dose ($e^- Ǎ^{-2}$)	20
Resolution (Ǎ)	4.2
Map Sharpening B-factor ($Ǎ^2$)	-175
**Model Refinement**	
Fo-Fc Correlation	0.78
Protein atoms	4163
R.m.s.d., bonds (Ǎ)	0.01
R.m.s.d., angles (°)	1.05
Clashscore, all atoms (percentile)	7.01
Rotamer Outliers (%)	0.0
Ramachandran outliers (%)	0.0

Supporting Figures.

Supporting Figure S1

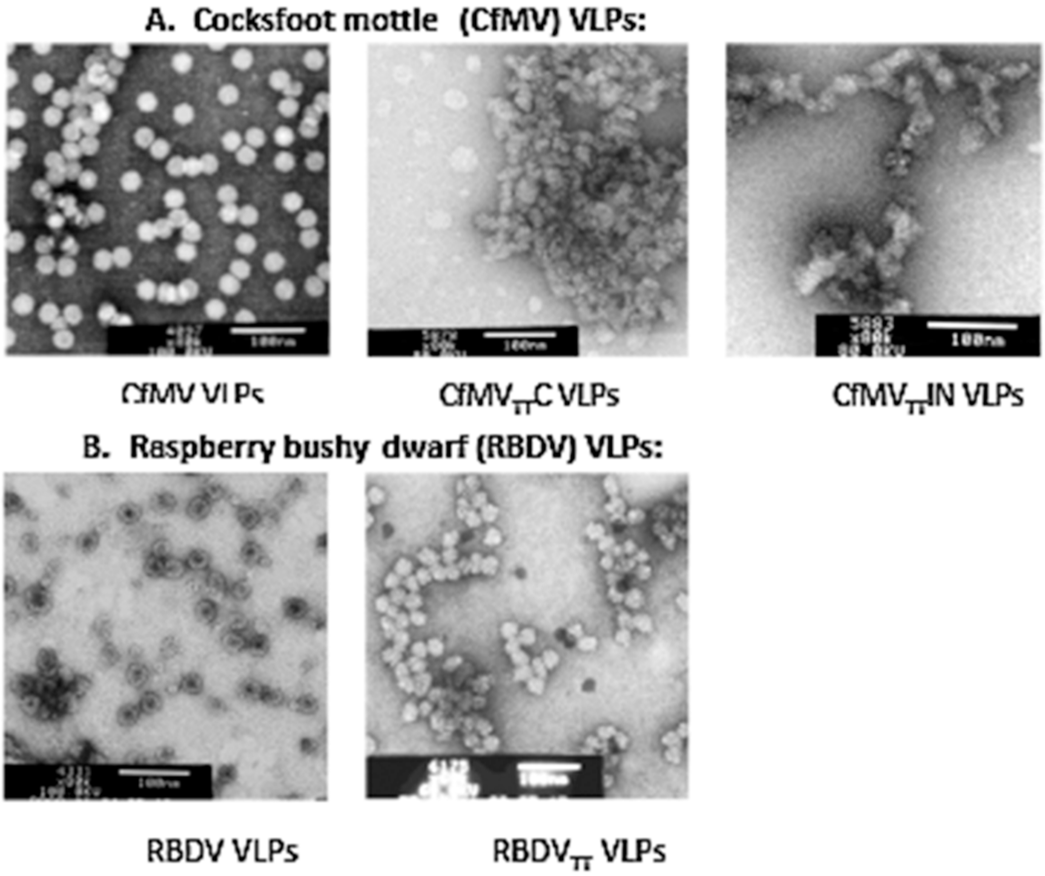

Figure S1. Electron microscopy pictures of CfMV VLP – wild type (A), after introducing TT epitope at C-terminus, which is located close to virion surface (B) and as internal insertion in a surface-located loop (C). Electron microscopy pictures of RBDV VLP – wild type (A), after replacing the original AA by TT-epitope based on AA sequence similarity (B) and storage at $+4^{\circ}$C and -20°C.

The parent CfMV three-dimensional (3D) structure is known (PDB No. 1NG0). We introduced TT-epitope at the C-terminus, which is located close to virion surface and as internal insertion in a surface-located loop. EM analysis suggest that both insertions prevent CfMV VLP formation. RBDV 3D structure is not known.

For RBDV, we replaced the original AA by TT-epitope based on AA sequence similarity. The replacement did not influence the VLP formation. However, $RBDV_{TT}$ VLPs were unsuitable for platform development due to strong aggregation of particles during storage at $+4^{\circ}$C and -20°C.

Supporting figure S2

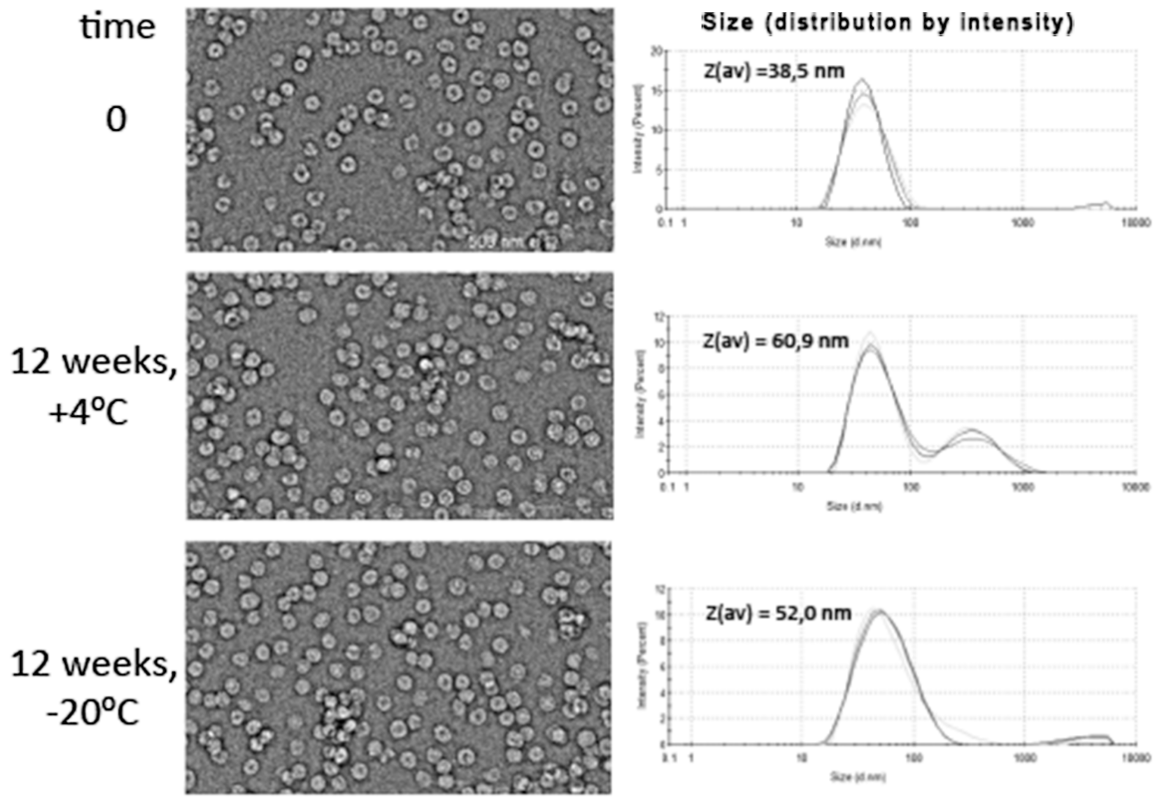

Supplement Figure 2. Stability assessement for integrity and aggregation of CMV$_{TT}$ particles. CMV$_{TT}$ VLPs: particle integrity, stability, and aggregation assay. CMV$_{TT}$ VLPs were produced as described in Materials and Methods. CMV$_{TT}$ particle solution in 5 mM sodium phosphate, 2 mM EDTA, pH 7.0 (1.5 mg/ml) was sterilized using 0.2μ filter and incubated at +4°C or -20°C. Left column – electron microscopy (EM) images of CMV$_{TT}$ VLPs at the beginning of experiment (time «0» sample) and after 12 week storage at different temperatures; Right column – dynamic light scattering (DLS) analysis of CMV$_{TT}$ VLPs (three subsequent analysis runs). Z(av) - mean hydrodynamic size of CMV VLPs (in nanometers). According to EM analysis, VLPs can be stored at different temperatures without significant particle disassembly and aggregation. However, DLS analysis suggest that CMV VLPs tend to aggregate at +4°C

Supporting Figure S3.

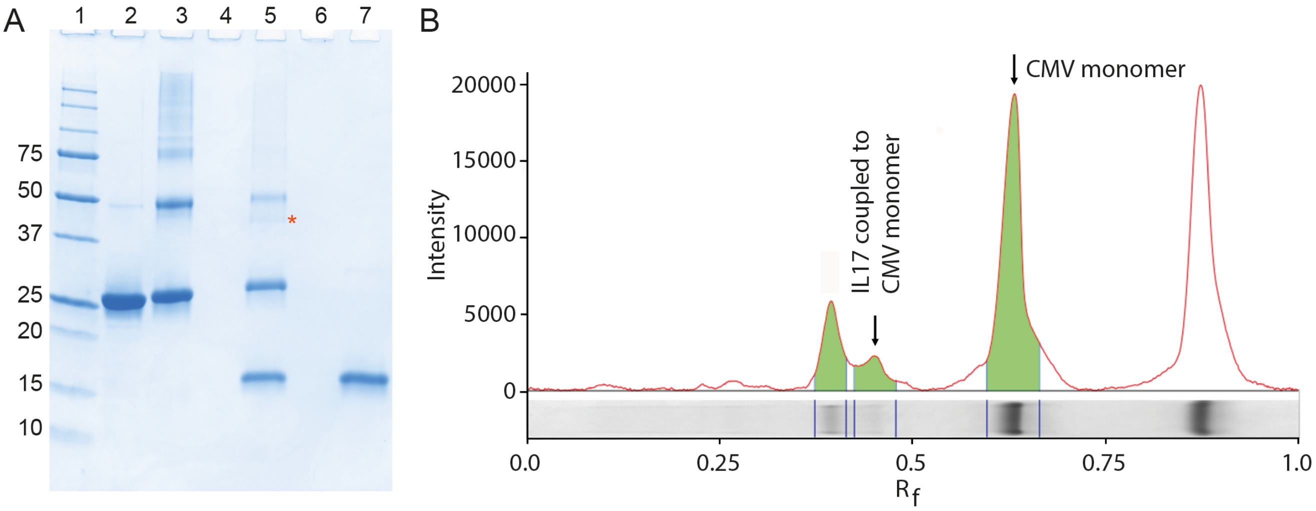

Band Label	MW	No. of coupled IL17 molecules	Net intensity (NI)	NI/ MW	Relative amount (RA) ((NI/ MW)/ Sum)	Epitope Density Σ (RA x No. IL17 molecules)	Coupling efficiency (%)
CMV monomer	24.5	0	65495434	2673283	0.923	0	
IL17 coupled to CMV monomer	40.8	1	9149902	224262	0.077	0.077	
Sum (Σ)				2897545		0.077	8

Figure S3. Analysis of the recombinant mIL17-CMV$_{TT}$ vaccine. (A) Coomassie stained SDS-PAGE run under reducing and denaturing conditions (left). Gel loaded left-right as follows: 1-MW marker (Bio-rad, Precision Plus protein standards unstained; 2-CMV$_{TT}$ VLP; 3-CMV$_{TT}$-SMPH derivatised VLP; 5-CMV$_{TT}$-IL17 conjugated vaccine (conjugated band highlighted with red asterisk); 7 -IL17 protein; lanes 4 and 6 empty. Samples derived from the same experiment and gels/blots were processed in parallel. (B) Graphical representation of densitometry analysis of lane 5 (CMV$_{TT}$-mIL17 vaccine sample). The density (net intensity, NI) of each CMV$_{TT}$ band was determined using Image Lab (version 5) software (Bio-Rad Laboratories). Using MW of 24.5 kDa for monomeric CMV$_{TT}$ and 40.8 kDa for IL17- CMV$_{TT}$ the relative amount (RA) of monomer CMV$_{TT}$ to monomer plus one antigen (mIL17-CMV$_{TT}$) as a proportion of the total for both bands was calculated. For a single conjugated protein this equates to the epitope density, which expressed as a percentage estimates ~8% of CMV$_{TT}$ corresponds to mIL17 conjugated form (red arrow). Calculated values for (B) are displayed in the table below the lane densitometry profile.

Supporting Figure S4

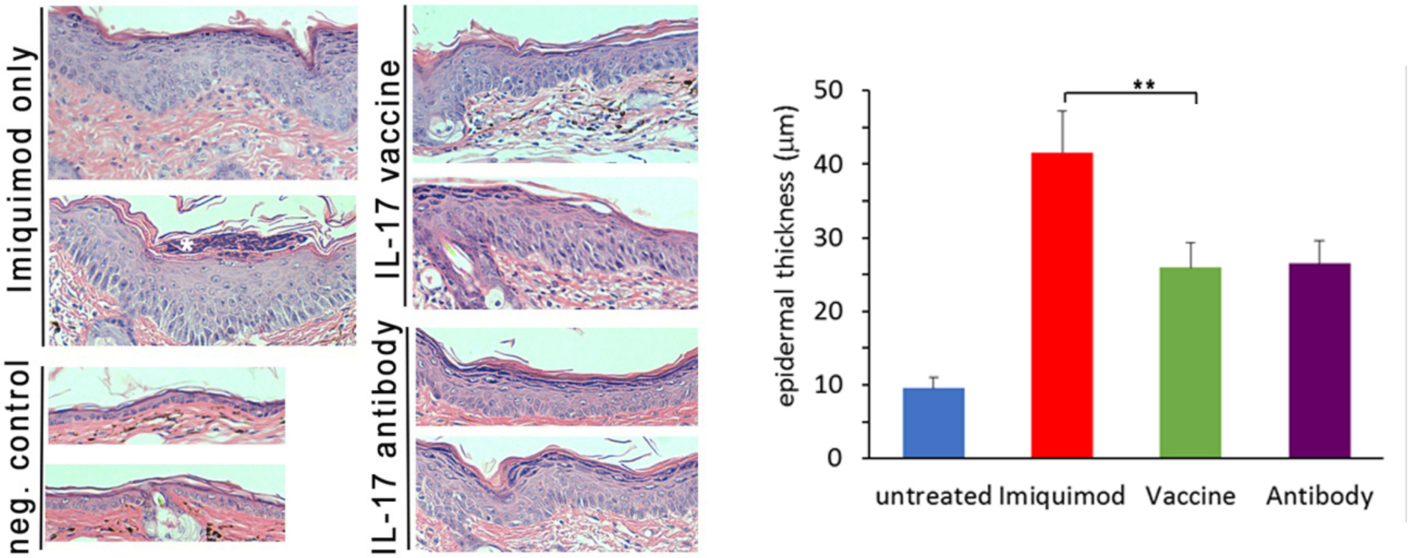

The efficacy of anti-IL17A vaccine compared to anti-IL17A monoclonal antibody on imiquimod-induced psoriasis like disease on dorsal skin of mice. Conditions and dosing were identical to those described in figure 4. Data shown represent dorsal skin samples (left), as well as overall epidermal thickness (average +/- s.d.) for n=5 mice per group. ** $p < 0.01$ in a two-sided T-test.

Supporting figure S5

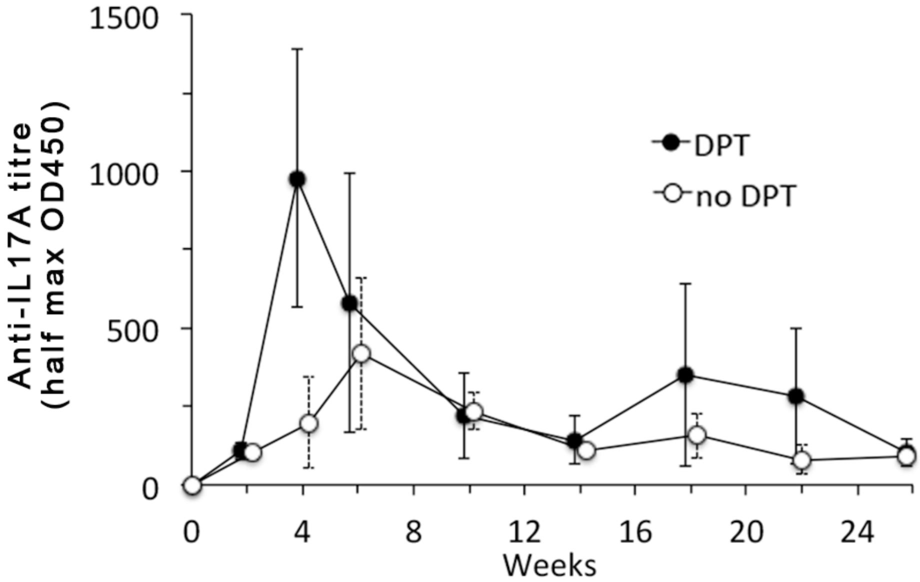

The effect of DPT-pre-vaccination on anti-IL17A-IgG titers induced in young C57B/6j female mice by sub-optimal dosing with anti-IL17 vaccine. Conditions shown are identical to those described in figure 4, except that mice were 10 weeks old at the start of pre-vaccination.

Supporting figure S6

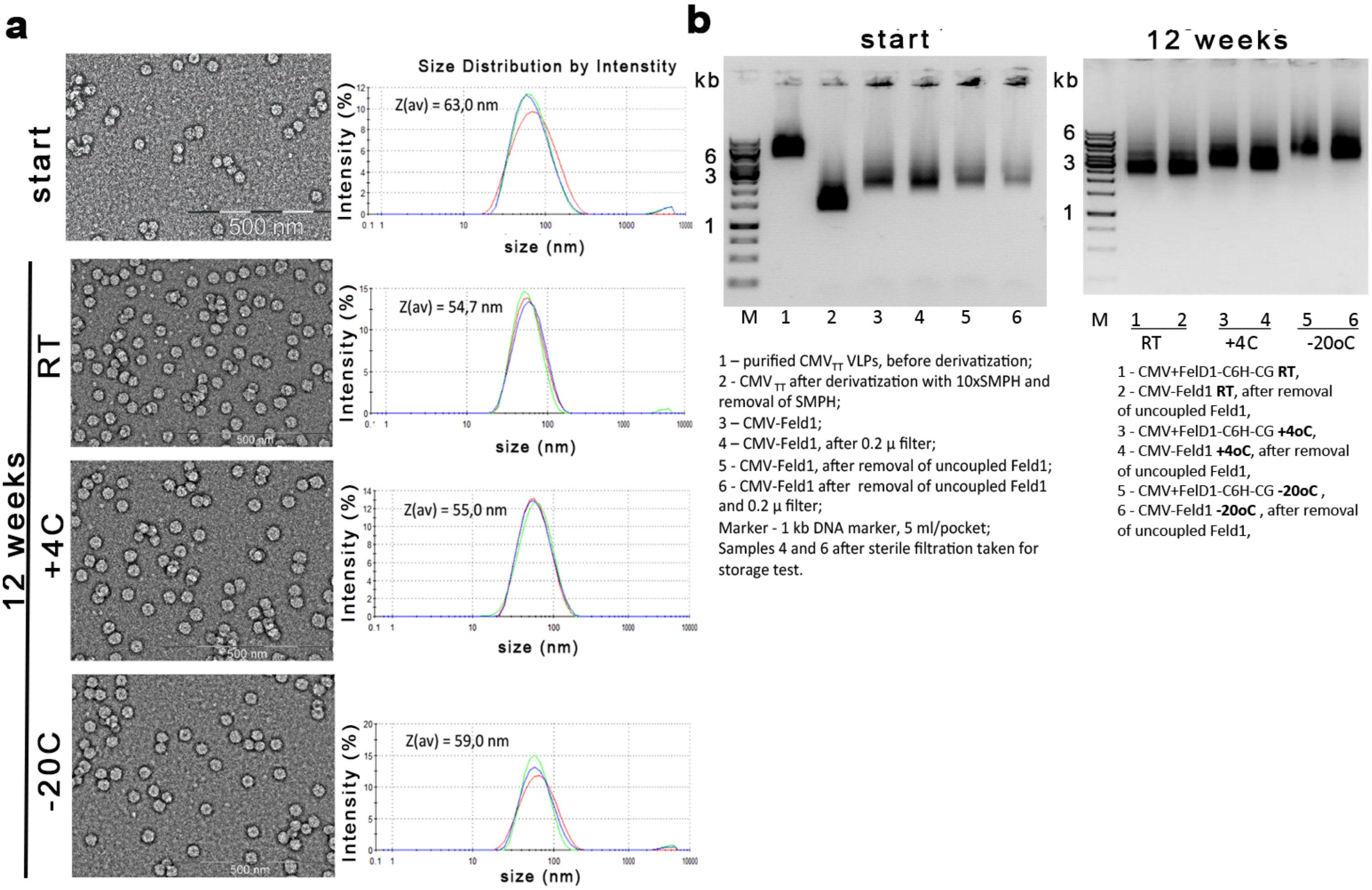

Stability assay for particle integrity and aggregation of the CMV-Feld1 vaccine. (A) Left – electron microscopy (EM) images of CMV-Feld1 vaccine at the beginning of experiment and after 12 week storage at different temperatures as shown; right - dynamic light scattering (DLS) analysis of CMV-Feld1 vaccine. Z(av) - mean hydrodynamic size of vaccine particles (in nanometers). (B) column – agarose gel analysis of CMV-Feld1 vaccine. Vaccine VLPs (6 µl, 1.0 mg/ml) were mixed with DNA Loading buffer and analyzed in 0.8% agarose/TBE buffer. Samples derived from the same experiment and gels/blots were processed in parallel. Samples loaded shown below each gel.
