# Supplementary figures and images for: Incorporation of tetanus-epitope into virus-like particles achieves vaccine responses even in older recipients in models of psoriasis, Alzheimer’s and cat allergy
Source: NPJ Vaccines. 2017 Oct 23;2:30. doi: 10.1038/s41541-017-0030-8 (PMC5653761; doi:10.1038/s41541-017-0030-8)

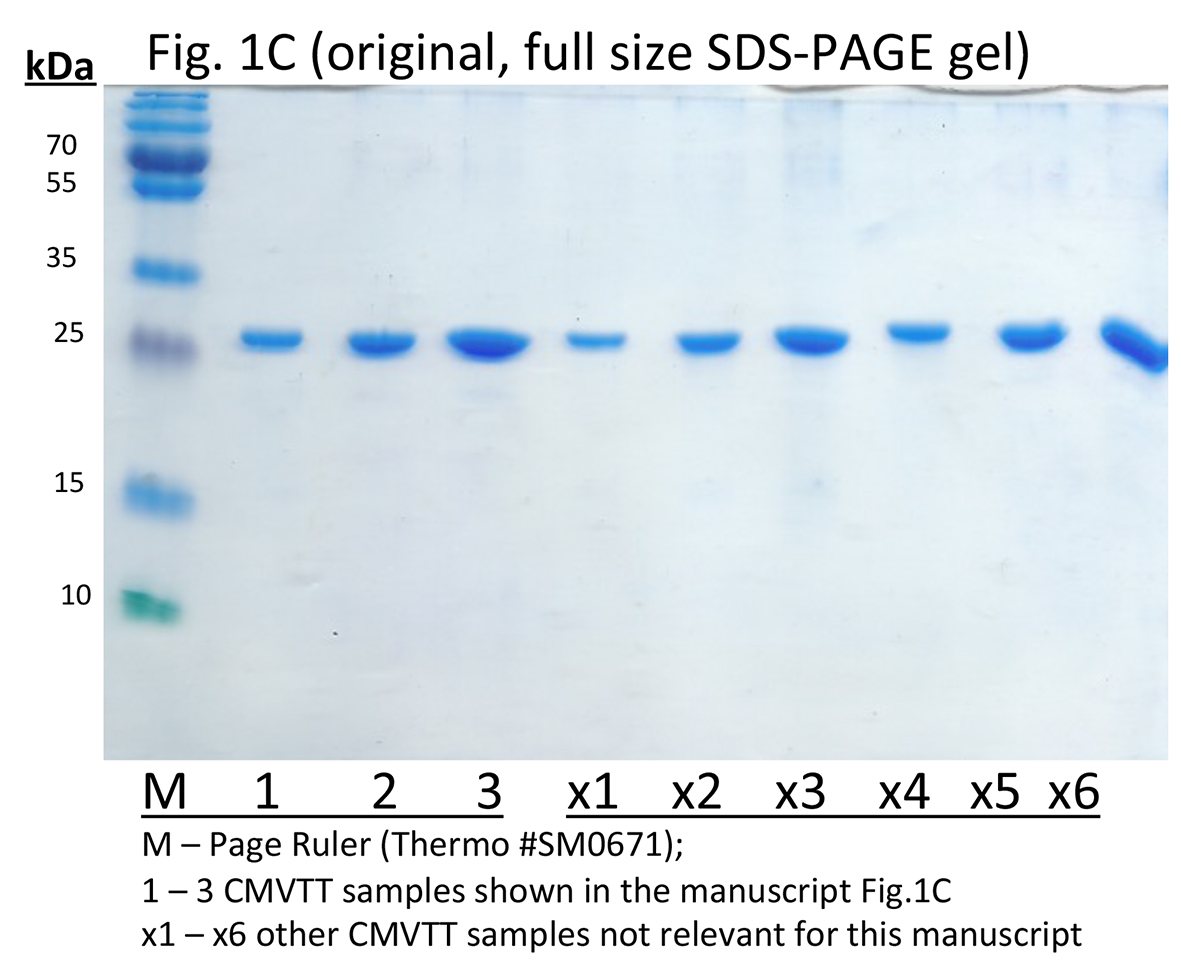

Supplement: Supplementary file 2 — Original gel files Fig1c [file 41541_2017_30_MOESM2_ESM.jpg]

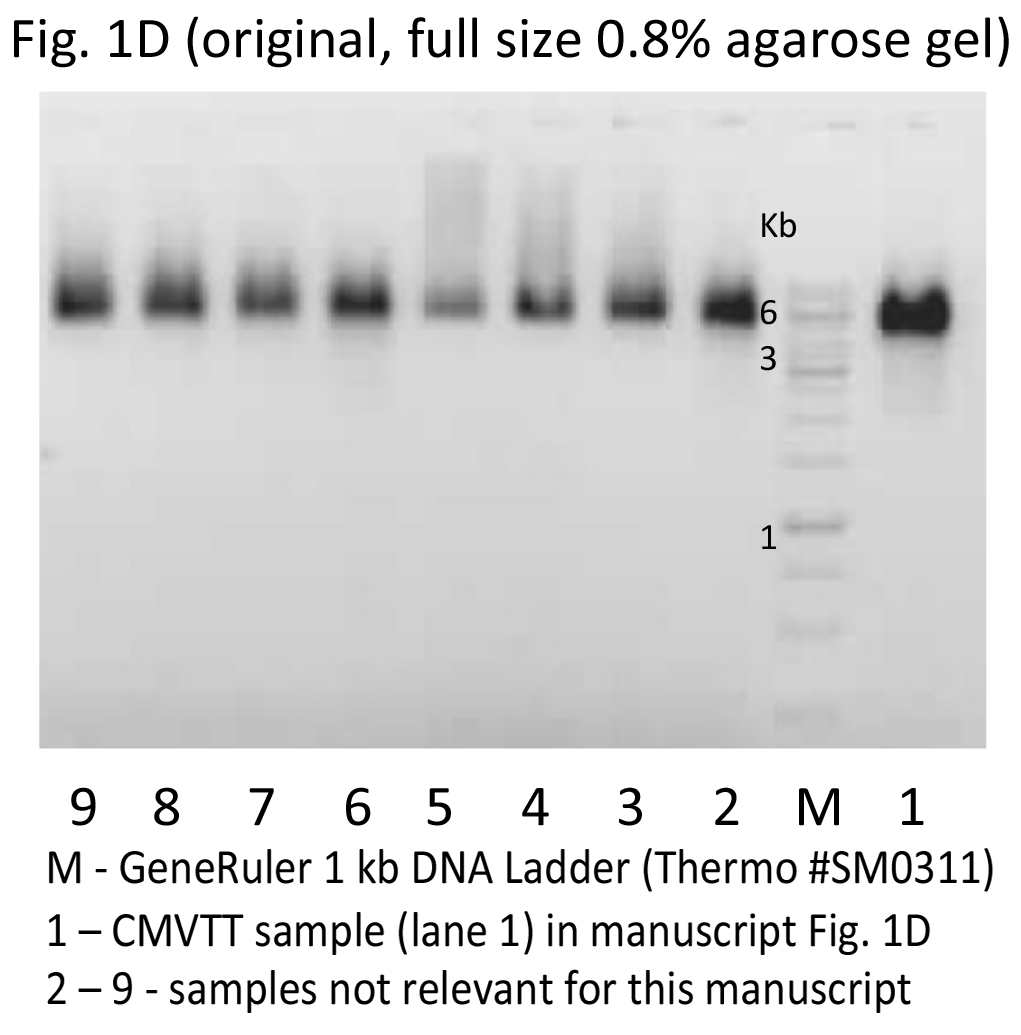

Supplement: Supplementary file 3 — Original gel file Fig1D [file 41541_2017_30_MOESM3_ESM.jpg]

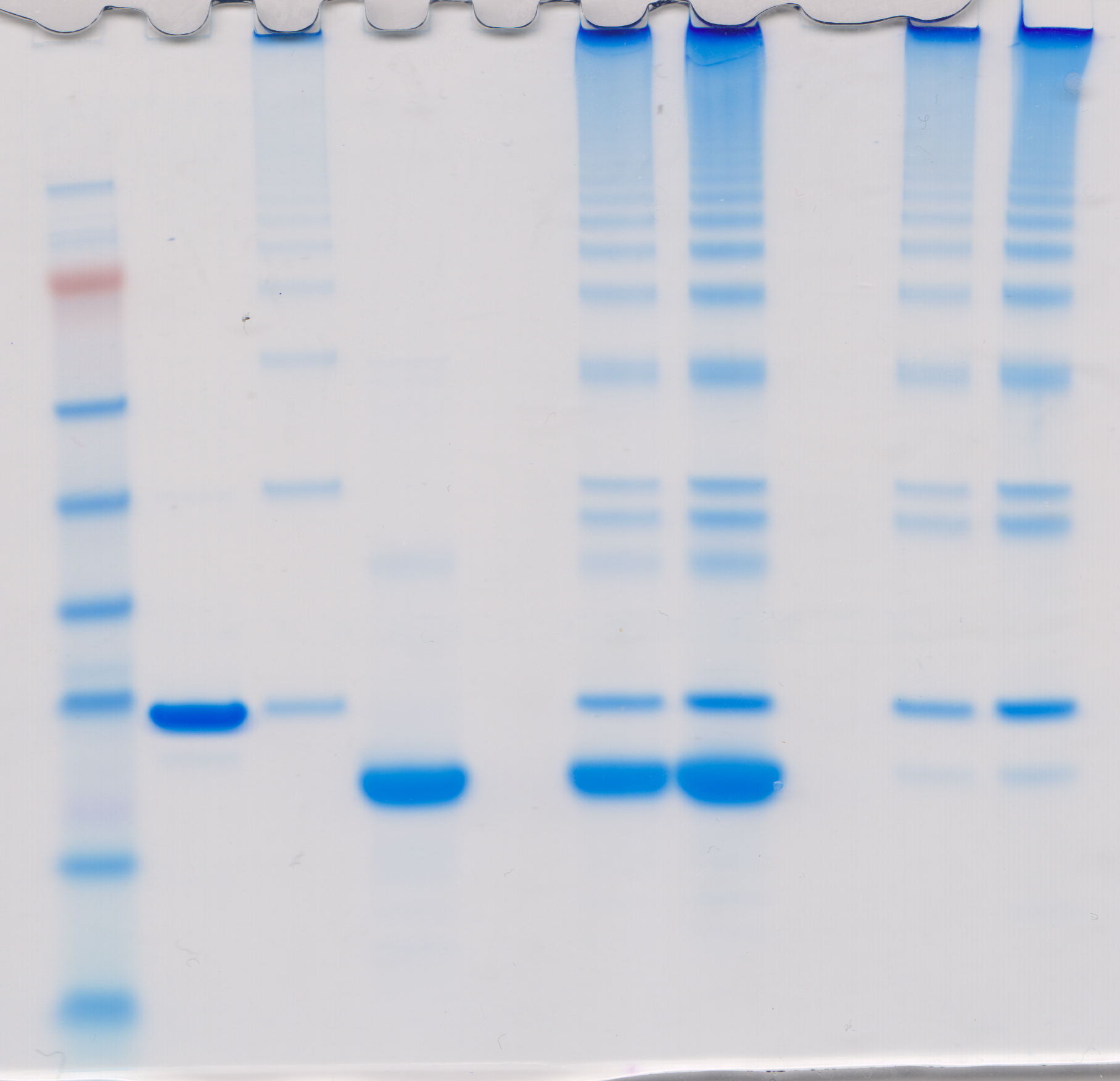

Supplement: Supplementary file 4 — Full length gel blot Fig 5A [file 41541_2017_30_MOESM4_ESM.jpg]

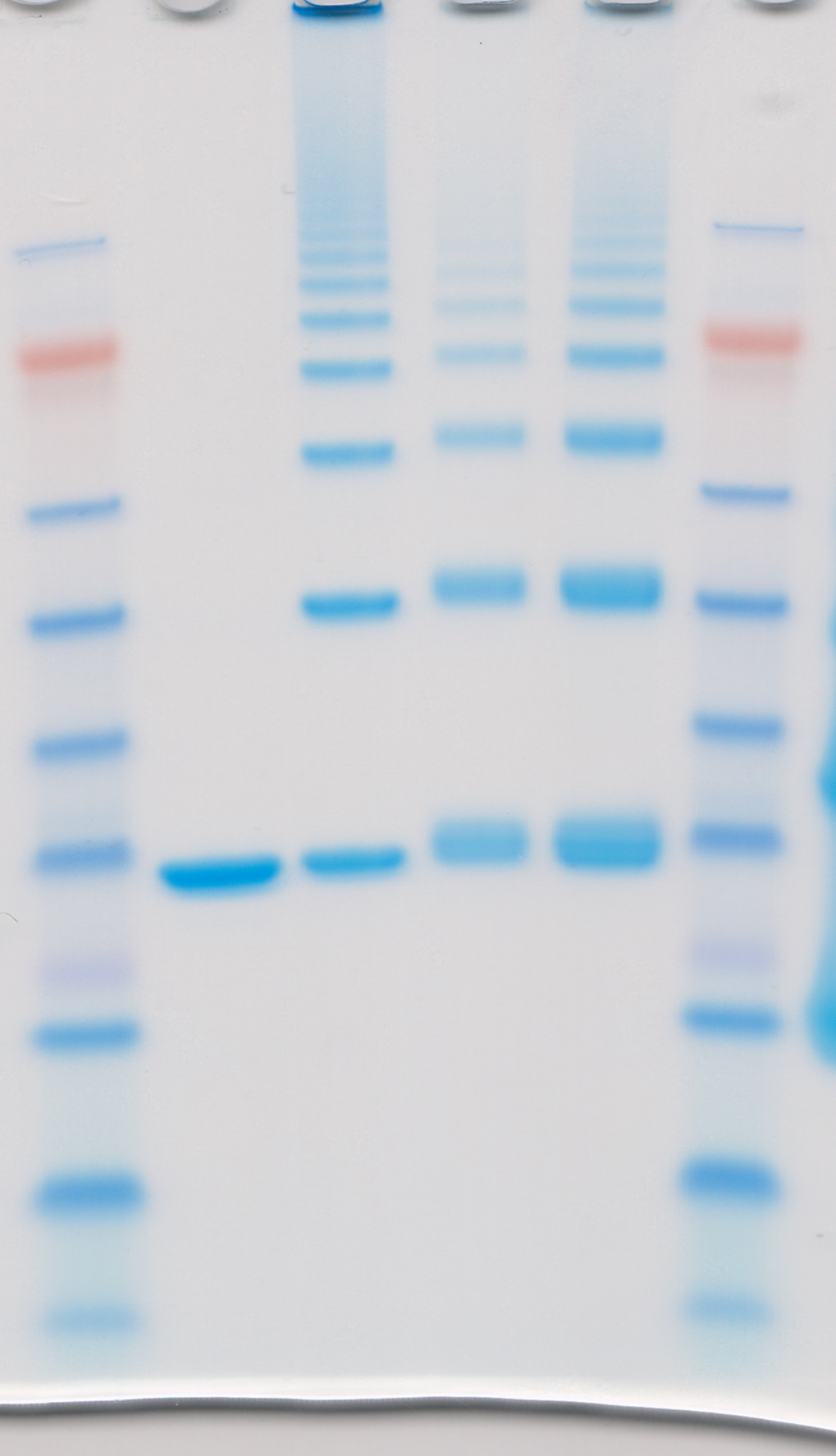

Supplement: Supplementary file 5 — Full length gel blot Fig 6A_left panel [file 41541_2017_30_MOESM5_ESM.jpg]

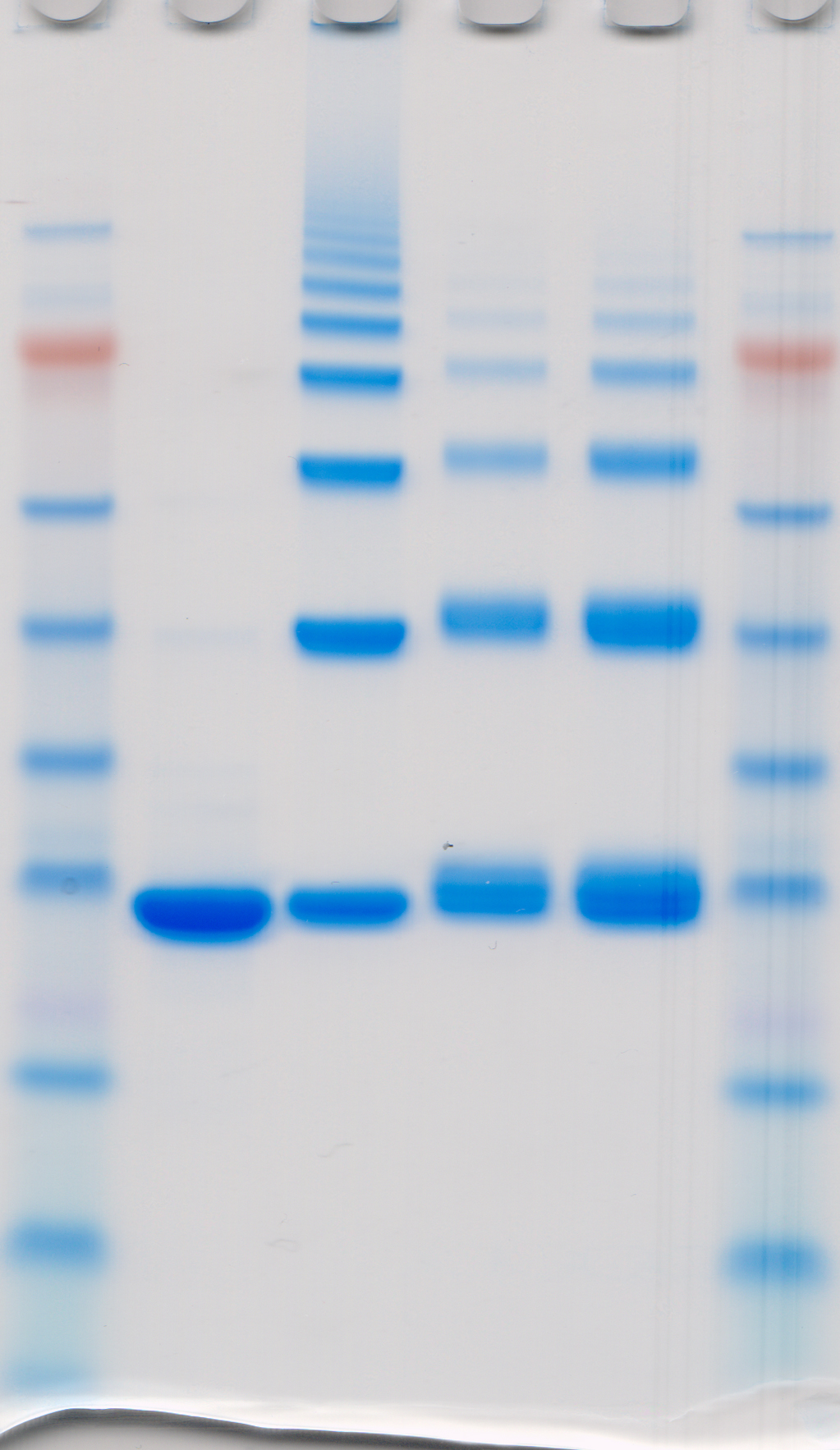

Supplement: Supplementary file 6 — Full length gel blot Fig 6A_right panel [file 41541_2017_30_MOESM6_ESM.jpg]

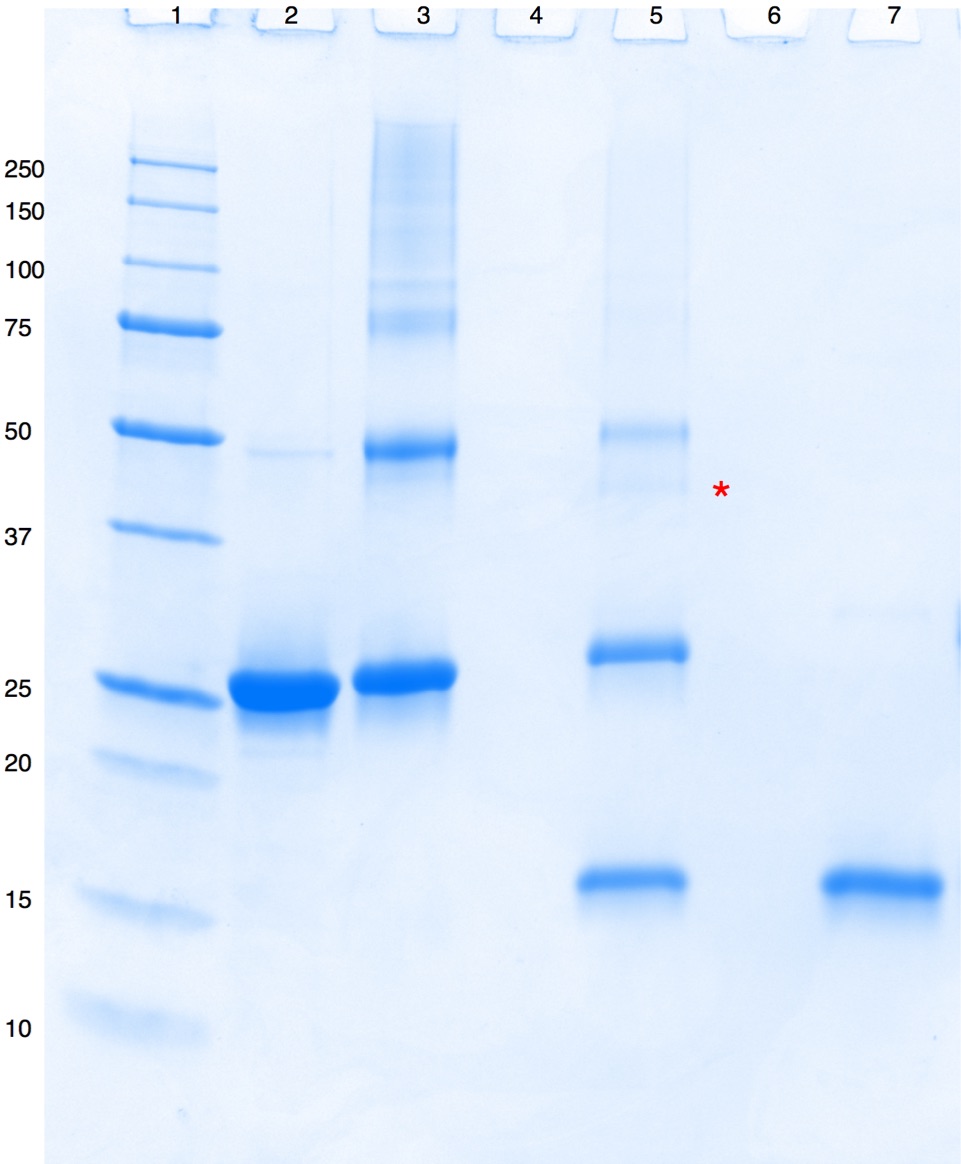

Supplement: Supplementary file 8 — Original gel file FigS3a [file 41541_2017_30_MOESM8_ESM.jpg]

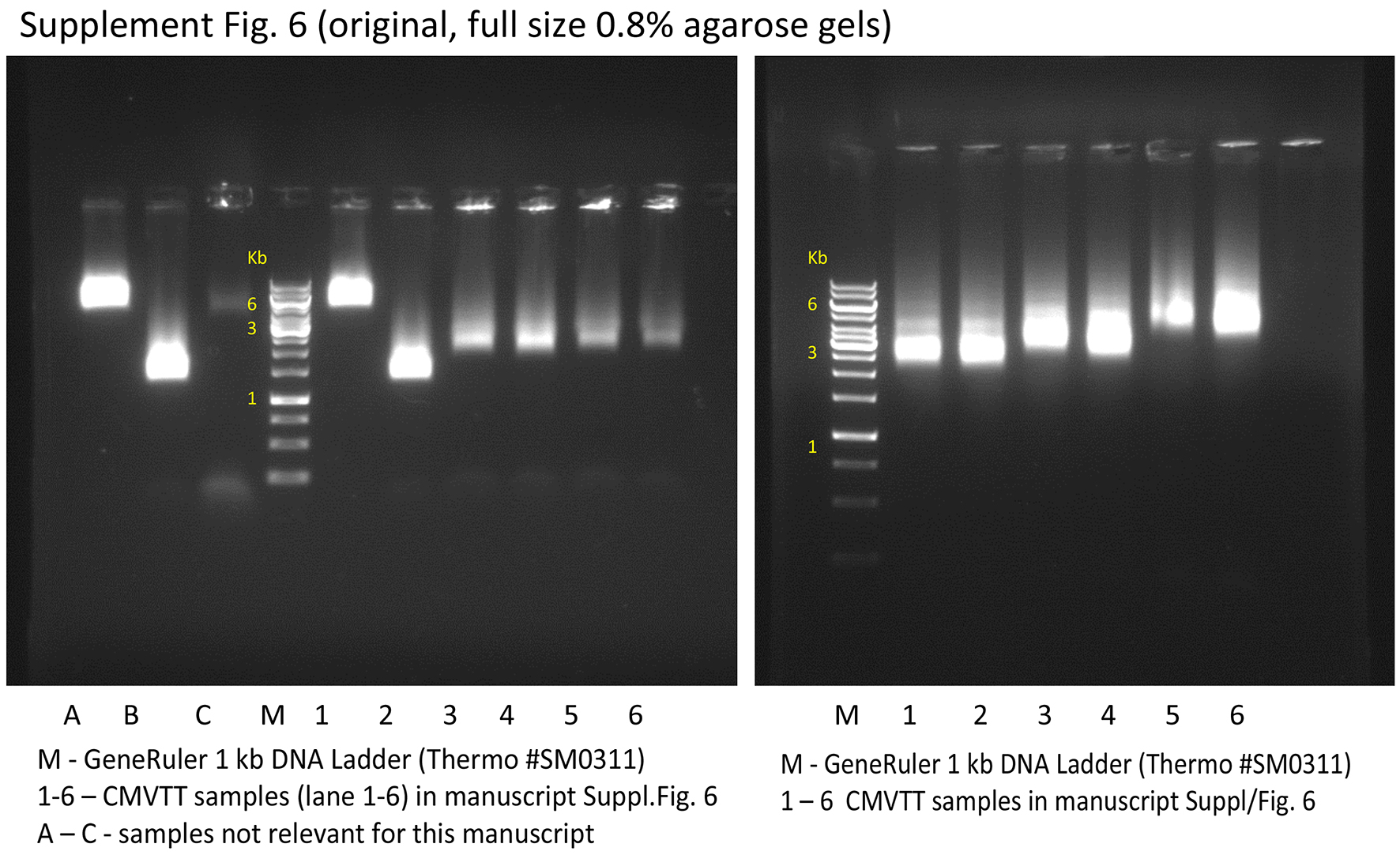

Supplement: Supplementary file 9 — Original gel file FigS6 [file 41541_2017_30_MOESM9_ESM.jpg]
