# Supplementary material for: Incorporation of tetanus-epitope into virus-like particles achieves vaccine responses even in older recipients in models of psoriasis, Alzheimer’s and cat allergy
Source: NPJ Vaccines. 2017 Oct 23;2:30. doi: 10.1038/s41541-017-0030-8 (PMC5653761; doi:10.1038/s41541-017-0030-8)
Supplement: Supplementary file 7 — Mol weight marker for Figure 5A, 6A [file 41541_2017_30_MOESM7_ESM.pdf]

SeeBlue® Plus2 Pre-Stained Protein Standard

novex® by life technologies™

Publication Part No. LC5925.pps **Publication No.** MAN0007923 **Rev** B.0

**Package contents**	**Catalog Numbers**<br>LC5925	**Size:**<br>500 µL
**Storage conditions**	Store at 2°C to 8°C.	
**Required materials**	▪ Polyacrylamide gel(s)<br>▪ Electrophoresis apparatus for protein analysis	
**Selection guide**	Pre-Stained Protein Standards<br>Go online to view related products.	
**Product description**	▪ SeeBlue® Pre-Stained Protein Standard allows you to accurately determine molecular weight ranges and evaluate western transfer efficiency during electrophoresis.<br>▪ This standard consists of 10 pre-stained protein bands (8 blue and 2 contrasting colors) ranging in molecular weight from ~3–200 kDa.<br>▪ Storage buffer: Tris-HCl, Formamide, SDS, and Phenol Red.	
**Important guidelines**	▪ Use with NuPAGE®, Tris-Glycine, and Tricine gels. Click here to view our online Protein Gel Selection Guide.<br>▪ DO NOT apply heat or add a reducing agent. SeeBlue® Plus2 Pre-Stained Standard is supplied ready-to-use.	
**Online resources**	Visit our product page for additional information and protocols. For support, visit www.lifetechnologies.com/support.	

For Research Use Only. Not for use in diagnostic procedures.

18 March 2014

SeeBlue® Plus2 Pre-Stained Protein Standard Protocol

Protocol

1. Thaw the standard at room temperature.
2. Vortex gently to ensure the solution is homogeneous.
3. Load the standard on the gel (see table for recommended volumes).

Gel Type	Applicaton	
	Electrophoresis	Blotting
Mini gel (1.0-mm thick)	10 µL	3 µL
Mini gel (1.5-mm thick)	20 µL	5 µL
Midi gel (1.0-mm thick)	20 µL	7 µL
Midi gel (1.5-mm thick)	30 µL	10 µL

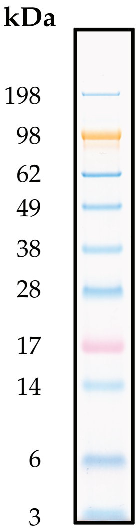

10 µL of SeeBlue® Plus2 Pre-Stained Protein Standard on a NuPAGE® 10% Bis-Tris Gel with MES SDS Running Buffer

Limited product warranty

Life Technologies Corporation and/or its affiliate(s) warrant their products as set forth in the Life Technologies' General Terms and Conditions of Sale found on Life Technologies' website at **www.lifetechnologies.com/termsandconditions**. If you have any questions, please contact Life Technologies at **www.lifetechnologies.com/support**.

Important licensing information

This products may be covered by one or more Limited Use Label Licenses. By use of this product, you accept the terms and conditions of all applicable Limited Use Label Licenses.

Disclaimer

LIFE TECHNOLOGIES CORPORATION AND/OR ITS AFFILIATE(S) DISCLAIM ALL WARRANTIES WITH RESPECT TO THIS DOCUMENT, EXPRESSED OR IMPLIED, INCLUDING BUT NOT LIMITED TO THOSE OF MERCHANTABILITY, FITNESS FOR A PARTICULAR PURPOSE, OR NON-INFRINGEMENT. TO THE EXTENT ALLOWED BY LAW, IN NO EVENT SHALL LIFE TECHNOLOGIES AND/OR ITS AFFILIATE(S) BE LIABLE, WHETHER IN CONTRACT, TORT, WARRANTY, OR UNDER ANY STATUTE OR ON ANY OTHER BASIS FOR SPECIAL, INCIDENTAL, INDIRECT, PUNITIVE, MULTIPLE OR CONSEQUENTIAL DAMAGES IN CONNECTION WITH OR ARISING FROM THIS DOCUMENT, INCLUDING BUT NOT LIMITED TO THE USE THEREOF.

© 2014 Thermo Fisher Scientific Inc. All rights reserved. All trademarks are the property of Thermo Fisher Scientific and its subsidiaries unless otherwise specified.

life technologies™

For support, visit www.lifetechnologies.com/support.
